# Quantitative analysis of CRISPR/Cas9-mediated provirus deletion in blue egg layer chicken PGCs by digital PCR

**DOI:** 10.1038/s41598-022-19861-7

**Published:** 2022-09-16

**Authors:** Stefanie Altgilbers, Claudia Dierks, Sabine Klein, Steffen Weigend, Wilfried A. Kues

**Affiliations:** 1 Department of Biotechnology, Stem Cell Physiology, Institute of Farm Animal Genetics, Friedrich-Loeffler-Institut, 31535 Neustadt, Germany; 2 Department of Breeding and Genetic Resources, Institute of Farm Animal Genetics, Friedrich-Loeffler-Institut, 31535 Neustadt, Germany

**Keywords:** Biotechnology, Molecular biology

## Abstract

Primordial germ cells (PGCs), the precursors of sperm and oocytes, pass on the genetic material to the next generation. The previously established culture system of chicken PGCs holds many possibilities for functional genomics studies and the rapid introduction of desired traits. Here, we established a CRISPR/Cas9-mediated genome editing protocol for the genetic modification of PGCs derived from chickens with blue eggshell color. The sequence targeted in the present report is a provirus (EAV-HP) insertion in the 5’-flanking region of the *SLCO1B3* gene on chromosome 1 in Araucana chickens, which is supposedly responsible for the blue eggshell color. We designed pairs of guide RNAs (gRNAs) targeting the entire 4.2 kb provirus region. Following transfection of PGCs with the gRNA, genomic DNA was isolated and analyzed by mismatch cleavage assay (T7EI). For absolute quantification of the targeting efficiencies in homozygous blue-allele bearing PGCs a digital PCR was established, which revealed deletion efficiencies of 29% when the wildtype Cas9 was used, and 69% when a high-fidelity Cas9 variant was employed. Subsequent single cell dilutions of edited PGCs yielded 14 cell clones with homozygous deletion of the provirus. A digital PCR assay proved the complete absence of this provirus in cell clones. Thus, we demonstrated the high efficiency of the CRISPR/Cas9 system in introducing a large provirus deletion in chicken PGCs. Our presented workflow is a cost-effective and rapid solution for screening the editing success in transfected PGCs.

## Introduction

Recently, the CRISPR/Cas9 system (Clustered regularly interspaced short palindromic repeat/CRISPR-associated endonuclease Cas9) has become the most dominant and precise gene editing tool in a wide variety of species, including livestock animals^1–7^ such as chickens^8–14^. The most promising way to introduce CRISPR-mediated germline modifications and transmissions in chickens is through PGCs. These cells are the unipotent precursors of sperm and ova and circulate in the blood of early chicken embryos, subsequently migrating into the developing gonads^15^. The diploid PGCs can be isolated from blood or embryonic gonads, allowing enrichment in vitro under defined cell culture conditions, which have been continuously improved in recent years^16,17^. The re-transfer of in vitro genetically modified and pre-selected PGCs into chicken embryos is currently the most efficient way to produce chickens with specific gene knock-out or knock-in modifications^9,18^.

The CRISPR/Cas system is a ribonucleoprotein with RNA-mediated recognition of genomic DNA and is relatively straightforward to manufacture and use, compared to protein binding-based methods such as TALEN (Transcription activator-like Effector Nucleases) and zinc finger nucleases^19,20^. The programmable gRNA (20-nucleotide (nt) targeting sequence) directs the Cas9 endonuclease to a specific genomic location, where the enzyme induces a double-strand break. Two major repair pathways are activated by this double-strand break, non-homologous end joining (NHEJ) or homology directed repair (HDR)^21^. The former dominates, as it is active in all cell cycles^22^. The outcome is a non-homologous end-to-end linkage of the cut DNA strand, which is error-prone in the form of insertions and/or deletions (indels)^23^. Indels within a coding exon can lead to frameshift mutations or a premature stop codon which probably results in nonfunctional proteins. Since an HDR is only active in the S and G2 phase of the cell cycle, it occurs less frequently^21^. The specific partial sequence of the homologous sister chromatid or that of an introduced DNA template with homologous arms is used as a repair template.

In the past, a loss-of-function study with knock-out of the egg white gene ovomucoid was performed to produce eggs with low allergenicity^24^. In addition, knock-out of the myostatin (*MSTN*) gene and the G0/G1 switch gene 2 (*G0S2)* resulted in transgenic chickens with higher meat production and reduced abdominal fat deposition, respectively^14,25^. The introduction of a single amino acid deletion (W38) into the chicken Na^+^/H^+^ exchanger type 1 gene (*chNHE1*) resulted in chicken cells and living chickens resistant against avian leukosis virus subgroup J (ALV-J)^11,26^. A significant advance was made by creating a genetically sterile chicken line by introducing a suicide gene into the *DAZL* locus, which provides embryos without endogenous PGCs, making them suitable recipients for donor PGCs carrying genetic modifications^13^.

The application of CRISPR/Cas9-mediated gene editing in chickens is steadily increasing. However, there is limited data about how to realize large deletions in chicken PGCs and how to detect desired on-target mutations without introducing any marker gene. In general, it is still a challenge to introduce mutations into stem cells, especially into germ cell progenitors, since DNA damage often induces germ cell apoptosis^27–29^.

In this study, we demonstrate the elimination of the entire provirus (EAV-HP) insertion in the 5' flanking region of the *SLCO1B3* gene^30^. Insertion of the EAV-HP provirus on chromosome 1 of the chicken genome causes aberrant expression of SLCO1B3 in the chicken's shell gland, leading to increased deposition of bile salts such as biliverdin, which enter the shell gland and cause a blue shell phenotype^30,31^. This proviral insertion occurs in Araucana chickens, a Chilean domestic breed, but has also been found in Chinese blue layer breeds such as Dongxiang and Lushi^32^.

We tested the performance of a wildtype Cas9 and a high-fidelity variant of the endonuclease within the same cell clone. The high-fidelity variant used in this study carries four exchanged amino acids residues (N497, R661, Q695, Q926)^33,34^. These amino acids residues are thought to interact non-specifically with the phosphate backbone of the target DNA strand^34^. Exchange of amino acids at these positions seem to improve specificity and resulted in fewer off-target events compared with wildtype Cas9, although the exact mechanism is still under investigation^34–36^.

For testing on-target efficiency and exact quantification of mutated alleles, digital PCR assays were designed. We used a nanofluidic chip digital PCR, in which the DNA template is compartmentalized into many individual PCR reactions, resulting in end-point fluorescence measurement for each partition. It is therefore highly sensitive in detecting low frequencies of deletions and also allows absolute quantification of genome editing events^37^. Absolute quantification of each target is converted into a relative value, which indicates the ratio between the genetically modified content and a reference^38^.

## Results

### CRISPR-mediated deletions of the EAV-HP

**Figure 1:**
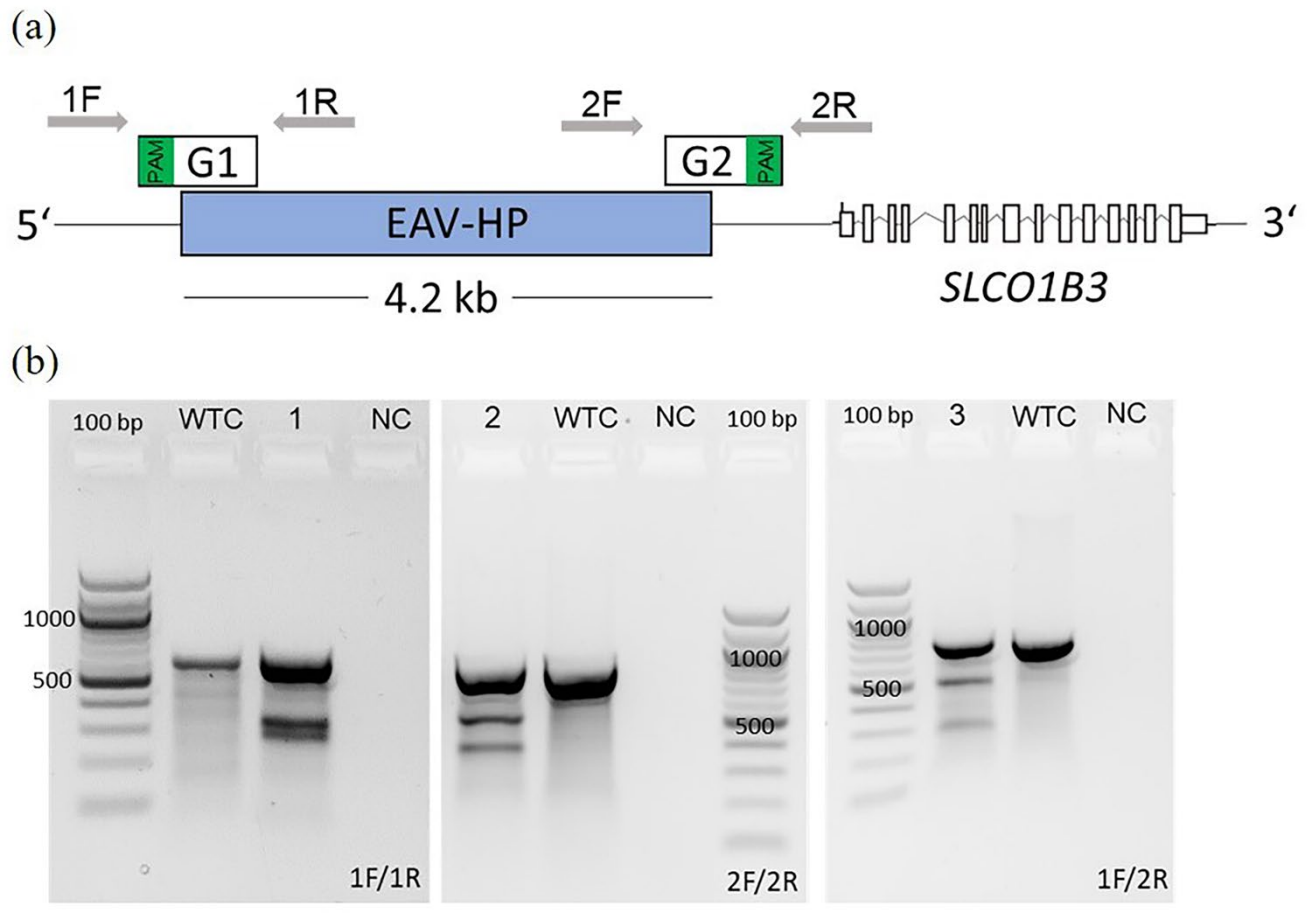
Assessment of CRISPR/Cas9-mediated mutations at the target site in homozygous blue-allele bearing PGC cell lines. (**a**) Schematic depiction of primer binding sites for amplification of the CRISPR/Cas9 target region on chromosome 1. The retroviral insertion is localized upstream of the *SLCO1B3* gene on the Araucana chicken chromosome 1. Guide RNA1 (G1) and guide RNA2 (G2) sequence specifically overlap partially with the provirus and the flanking sequences. The primer pairs 1F/1R and 2F/2R were used to amplify the specific target site of guide RNA 1 (G1) and guide RNA 2 (G2) respectively. Primer 1F and 2R were combined to detect cell clones with deletion of the provirus (786 bp amplicon). Green: PAM location (**b**) T7 endonuclease-I assay results of transfected PGCs (high-fidelity Cas9). Sample 1, 2 and 3 with additional bands of expected sizes on agarose gels, indicating gene editing events. WTC: wildtype control (DNA from PGCs electroporated without CRISPR plasmids), NC: negative control (water sample), 1: PGCs transfected with gRNA1 (G1), 2: PGCs transfected with gRNA2 (G2), 3: PGCs co-transfected with gRNA1 and gRNA2; uncropped versions of the agarose gel images were added to the Supplementary information (Fig. S11).

The EAV-HP target is located upstream of the *SLCO1B3* gene on chromosome 1 in Araucana chickens^30^. After testing several gRNAs near the insertion site which lacked efficiency to delete the entire provirus (Table S1), we finally identified a pair of gRNAs with high on-target specificity, causing a large deletion of approximately 4.2 kb in homozygous blue-allele bearing PGCs (Fig. 1a; Table S1). The 20 base pairs of each designed gRNA overlapped partially with the EAV-HP sequence and the flanking sequence of the chicken chromosome 1 (Fig. 1a). The protospacer adjacent motifs (PAM) and seed sequences of the gRNAs were located in the flanking region of the reference genome (Fig. 1a). Each gRNA was first tested separately in a single transfection experiment, followed by co-transfection of both guides to eliminate the entire EAV-HP provirus (4.2 kb). A gRNA was discarded if it did not complete the T7 assay positively or if the Sanger sequencing did not differ from the wildtype reference. The efficiency of genome editing with these gRNAs would be too low to perform the challenging single-cell dilution of PGCs.

The homozygous blue-allele bearing PGCs were transfected by electroporation with plasmids carrying the Cas9 endonuclease and the gRNA sequence on a single plasmid. One plasmid carrying a wildtype Cas9 and another plasmid carrying a high-fidelity variant of the endonuclease were tested. Three primer pairs were designed to amplify the specific target regions (Fig. 1a) to subsequently detect the CRISPR/Cas9-mediated mutations with a T7 endonuclease-I assay (T7EI). For the T7EI assays, PCR amplicons from DNA, which was isolated from the transfected PGCs, were used. Transfected PGCs were treated with puromycin to select for Cas9-transfected and resistance gene expressing cells. The results indicated cleavage of the heteroduplex DNA, resulting in additional bands on the agarose gel (Fig. 1b). This indicated on-target efficiency of the gRNAs of the CRISPR/Cas9 system. The additional bands were of expected size and non-transfected wildtype controls displayed no additional bands. As control for the PCR 1F/2R, we used a nullizygous blue-allele PGC line. Considering that we transfected homozygous blue-allele bearing PGCs, the appearance of an amplicon in PCR 1F/2R already confirmed the occurrence of a large deletion of the provirus within the tested cell population. PCR 1F/2R has not been established as a long-range PCR for amplification of the entire provirus, but results in a shorter amplicon only for larger deletions.

**Figure 2:**
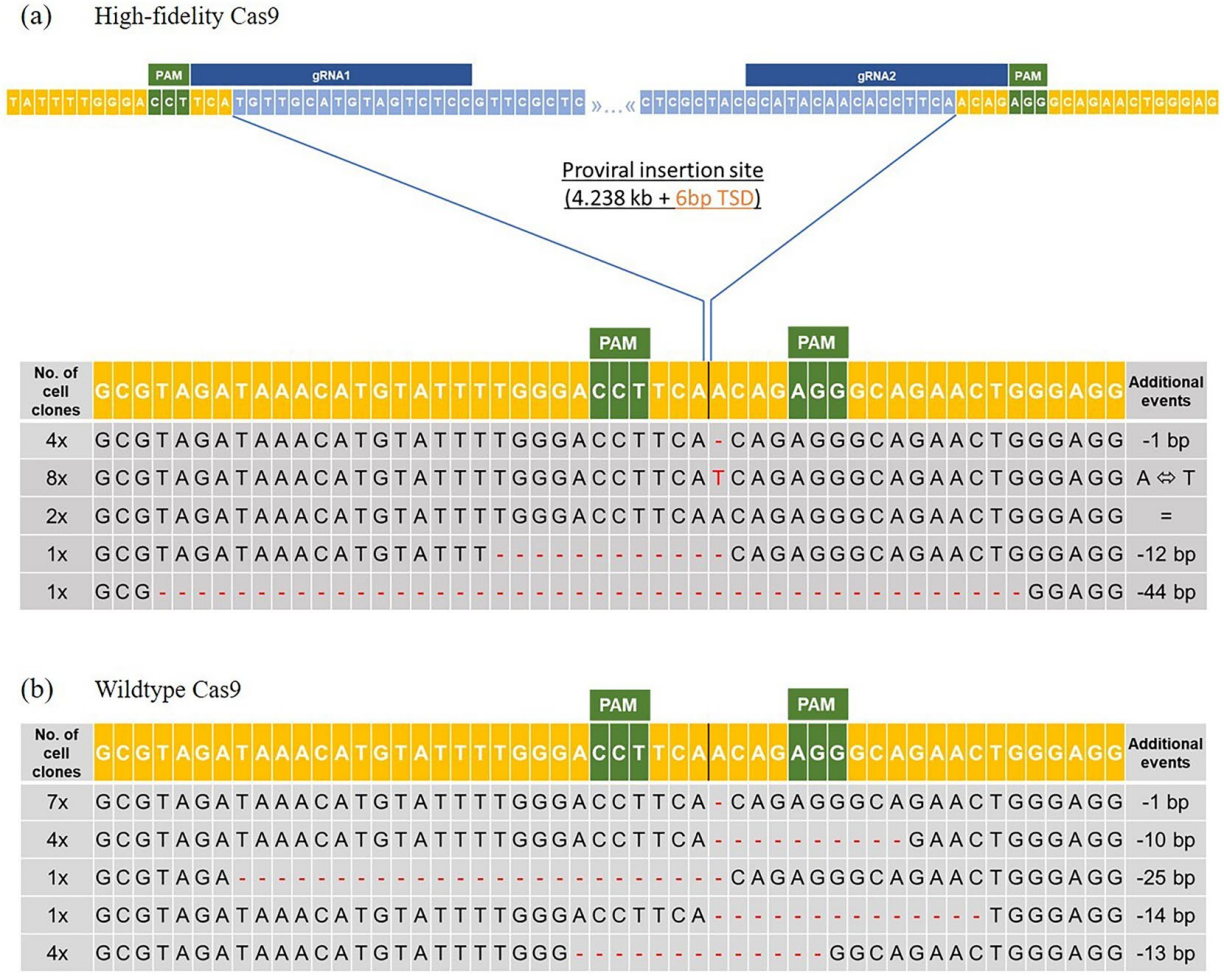
CRISPR/Cas9-mediated deletion events at the blue egg locus in Araucana crossbreed chicken PGCs. Yellow: Chromosome 1, light blue: EAV-HP retrovirus sequence, orange: 6 bp target-site duplication (TSD), *PAM* protospacer adjacent motif (green), dark blue: guide RNA. (**a**) Sanger sequencing of 16 single PCR clones (1F/2R) with deletion of the provirus insertion. Results are based on co-transfection of PGCs with gRNA1 and gRNA2 using a high-fidelity variant of the Cas9 endonuclease; (**b**) Sanger sequencing of 17 single PCR clones (1F/2R) with deletion of the provirus insertion. Results are based on co-transfection of PGCs with gRNA1 and gRNA2 using the standard Cas9 endonuclease.

The 1F/2R PCR product of each Cas9 variant was then sub-cloned and Sanger sequenced. The respective PCR single clone sequences were aligned against a corresponding Sanger sequence of a non-blue allele bearing White Leghorn chicken and numbers of different mutations were listed (Fig. 2). For the high-fidelity variant, 16 individual PCR clones (Fig. 2a) and for the wildtype Cas9 (Fig. 2b) 17 individual PCR clones were evaluated. For the wildtype Cas9, deletions of the EAV-HP and few adjacent bases were found, while for the high-fidelity variant, deletion of the EAV-HP and few adjacent bases, but also an almost seamless deletion event with a single base inversion (A/T), and a seamless EAV-HP deletion were detected. The results demonstrate that the entire EAV-HP sequence of 4238 bp could be eliminated in Araucana chicken PGCs, regardless of which Cas9 variant was used.

In addition, the 1F/1R and 2F/2R PCR products (high-fidelity Cas9 only) were sub-cloned and Sanger sequenced (Fig. S1). For gRNA1, guide-associated edits were found in eight of 12 sequenced PCR clones (66%) and for gRNA2 in 4 of 10 sequenced PCR clones (40%).

### Quantitative detection of mutated alleles using digital PCR

**Figure 3:**
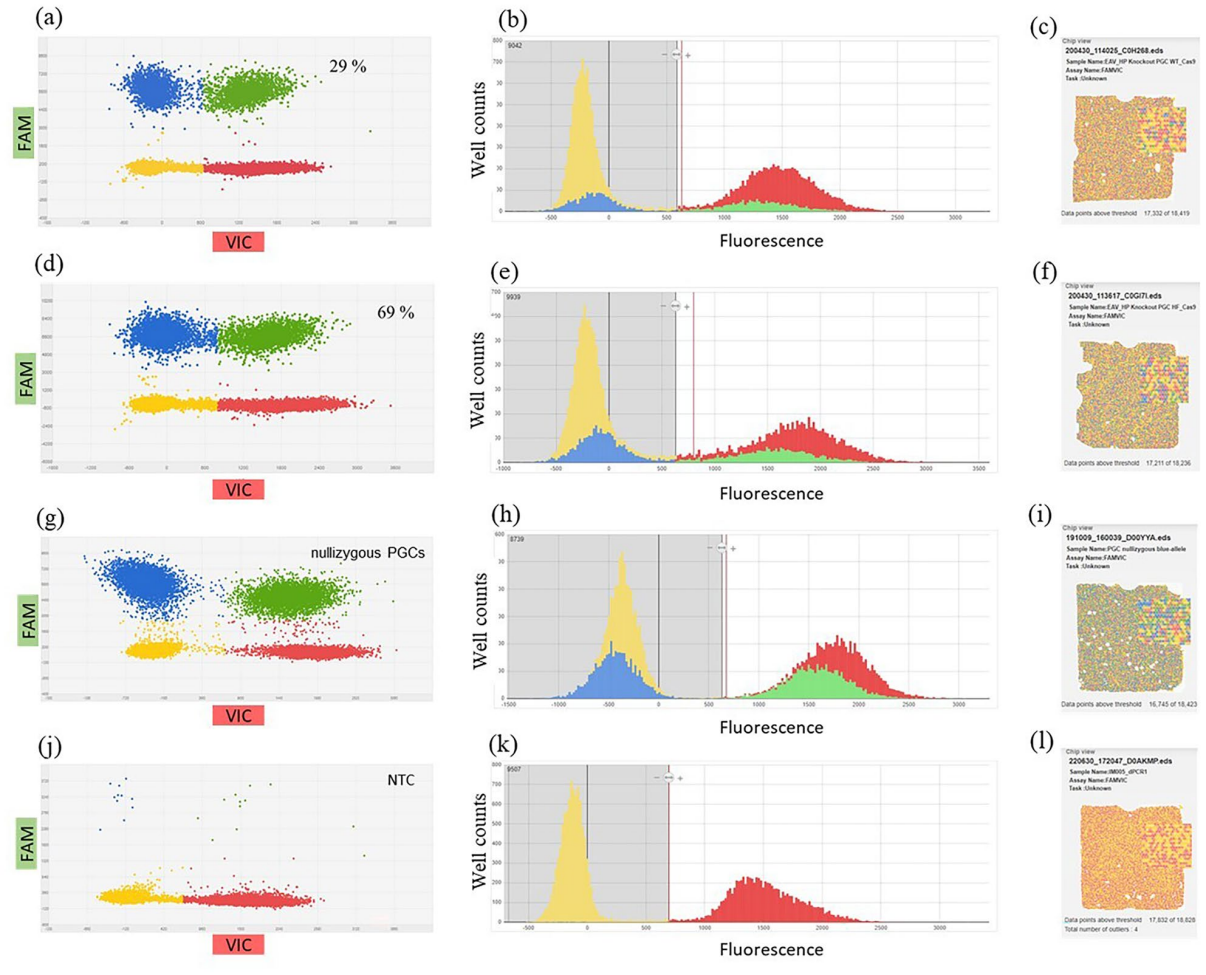
Digital PCR of nullizygous blue-allele PGCs, mixed knock-out cell populations and a non-target control (edited and non-edited cells, digital PCR 1). (**a,b,d,e,g,h,j,k**): blue: FAM reporter dye signal, red: VIC reporter dye signal, green: FAM/VIC reporter dye signals, yellow: no amplification (DNA-empty wells), VIC: reference assay (β-actin), FAM: digital PCR 1. (**a**) Two-dimensional scatterplot of digital PCR duplex assay (FAM/VIC) and (**b**) histogram from PGCs (knock-out approach in homozygous blue-allele bearing PGCs from Araucana chickens (mixed cell population), wildtype Cas9); (**c,f,i,l**) chip view by calls (uniformity). (**d**) scatterplot of digital PCR duplex assay (FAM/VIC) and (**e**) histogram from PGCs (knock-out approach in formerly homozygous blue-allele bearing PGCs from Araucana chickens (mixed cell population), high-fidelity Cas9); (**g**) scatterplot of digital PCR duplex assay (FAM/VIC) and (**h**) histogram from PGCs (control: nullizygous blue-allele bearing PGCs, no EAV-HP insertion on chr. 1); (**j**) scatterplot of digital PCR duplex assay (FAM/VIC) and (**k**) histogram from PGCs [*NTC* non-target control (FAM), homozygous blue-allele bearing PGCs].

**Figure 4:**
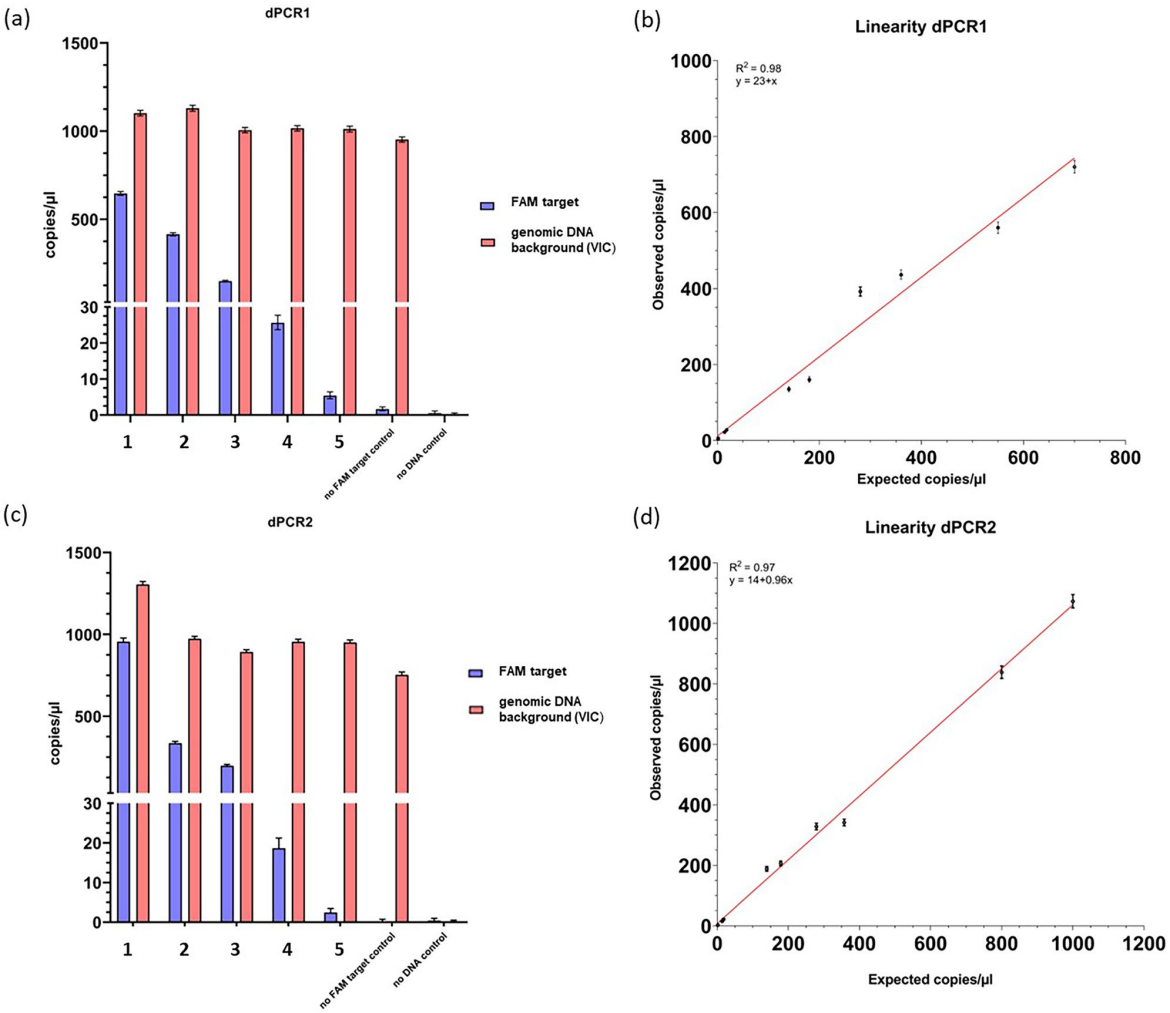
Quantitative nature of digital PCR1 and digital PCR2. Serial dilution of genomic DNA from two cell types: a knock-out single-cell clone (**a,b**) and homozygous-blue allele-bearing PGCs (**c,d**) at 650 to 5 copies/µl and 970 to 2 copies/µl per 14.5 µl reaction, respectively (FAM target). DNA was diluted in a constant background of genomic DNA (without FAM target) at 890 to 1300 copies/µl (**a,b**) and 990 to 1100 copies/µl (**c,d**) (VIC target). (**a**) Mean copies/µl of background genomic DNA (red bars) and diluted DNA (blue bars) for dPCR1, (**b,d**) expected and observed values of the dilution series (dPCR1 and dPCR2 results), data include two dilution series with different initial DNA concentrations. Error bars indicate 95% CI, (**b**) linearity of dPCR1 results, (*F*_1,8_ = 380.2, *p* < .001), (**c**) mean copies/µl of background genomic DNA (red bars) and diluted DNA (blue bars) for dPCR2, (**d**) linearity of dPCR2 results, (*F*_1,8_ = 239.5, *p* < .001).

Using the PCR 1F/2R the mutated alleles with deletion of the provirus were selectively amplified. Therefore, no quantification of the editing events in the transfected PGC population was possible. To quantify the number of mutant alleles accurately, a digital PCR assay (digital PCR 1) was developed to detect rare events. The assay was designed as duplex hydrolysis assay with a primer/probe pair binding the β-actin (*ACTB*) gene locus, which was set as biallelic copy number reference and labelled with VIC dye. A CRISPR/Cas9-mediated deletion of the provirus sequence led to the amplification of the shortened deleted sequence, making a fluorescence signal detectable for the FAM labelled primer/probe combination of the duplex assay (Fig. S2). The two-dimensional scatterplot illustrates that each partition can fall into one of three possible outcomes: partitions that contain (1) no DNA molecules (yellow), (2) a single DNA molecule (blue or red), or (3) more than a single DNA molecule, resulting in positive signals for both targets (green) (Fig. 3). The copy numbers of the target specific assay (FAM) were quantified in relation to the copy numbers of the β-actin assay (VIC). The copy number results of the digital PCR assay revealed that 29% of the mutant alleles were detectable following the use of the wildtype variant of the Cas9 endonuclease (Table 1, Fig. 3a–c). In the case of the high-fidelity Cas9 endonuclease, 69% of the mutant alleles (target/total) were detectable in a mixed cell population with wildtype alleles (Table 1, Fig. 3d–f). A nullizygous blue-allele PGC line was used as reference control, displaying almost equal copy numbers in the VIC and FAM channel (Table 1; Fig. 3g–i). Quantification of DNA from homozygous blue-allele bearing PGCs (no FAM target control) resulted in a false-positive rate for the FAM signal of 0.17% (1.68 copies/µl) (Table 1, Figs. 3j–l, 4a). Lower background fluorescence was observed when only the master mix without DNA (no DNA control) was loaded onto the chip (Table 1, Fig. 4a).

**Table 1:** Results of digital PCR 1 of nullizygous and knock-out PGCs.

Assay	Sample	Target/total (%)	Mean copies/µl
FAM	Nullizygous blue-allele bearing PGCs	100	880.2
VIC			862.6
FAM	EAV-HP knock-out PGCs (mixed population) HF-SpCas9	69	484.3
VIC			700.6
FAM	EAV-HP knock-out PGCs (mixed population) WT-SpCas9	29	300.9
VIC			859.9
FAM	Homozygous blue-allele bearing PGCs (no FAM target control)	0.17	1.6
VIC			952.4
FAM	No DNA control	–	0.5
VIC			0.08

To test the quantitative range of the digital PCR 1, we performed a serial dilution of a knockout single-cell clone, generated by limited dilution of transfected PGCs, in a constant background of chicken genomic DNA without binding site for the FAM-labeled probe (Fig. 4a). The observed copies per microliter (FAM) were plotted against the theoretical quantities (Fig. 4b). Simple linear regression analysis revealed high linearity with an R^2^ of 0.98. Quantification of FAM-positive partitions was detected in a range from 650 copies per microliter down to 5 copies per microliter at consistently high levels of background DNA.

Detailed digital PCR output data of the QuantStudio 3D AnalysisSuite software analysis are added as a supplementary Excel file.

### PGC cell clones with biallelic knock-out of the EAV-HP provirus

To further classify whether biallelic knock-out PGCs were generated by using the high-fidelity Cas9, limited dilutions of the transfected PGC population were performed. This resulted in PGC cell clones that were analyzed by PCR for the absence of the provirus sequence using the PCR primer pairing 1F/2R and 2F/2R (Fig. S3). Out of 23 PGC cell clones tested, 14 cell clones yielded no amplicon in PCR 2F/2R but an amplicon for PCR 1F/2R (Fig. S3), confirming that the provirus was deleted. The remaining nine clones still showed an amplicon in PCR 2F/2R (Fig. S3) and no amplicon for PCR 1F/2R (not shown), confirming that the provirus was still present. It turned out that only cell clones with a biallelic deletion of the provirus were established and no cells with a monoallelic deletion were found. Each biallelic cell clone was Sanger sequenced (Fig. S4) and aligned to the corresponding Sanger sequence of a White Leghorn chicken. The detection of three Araucana-specific SNPs in close proximity to the insertion site of the provirus additionally confirmed that the knock-out cell clones were formerly blue-allelic bearing Araucana crossbreed PGCs (Fig. S4). The different mutations detected in the individual PCR clones (high-fidelity Cas9) could also be found in the PGC cell clones after performing single cell dilution (Fig. 2a, Fig. S4).

**Figure 5:**
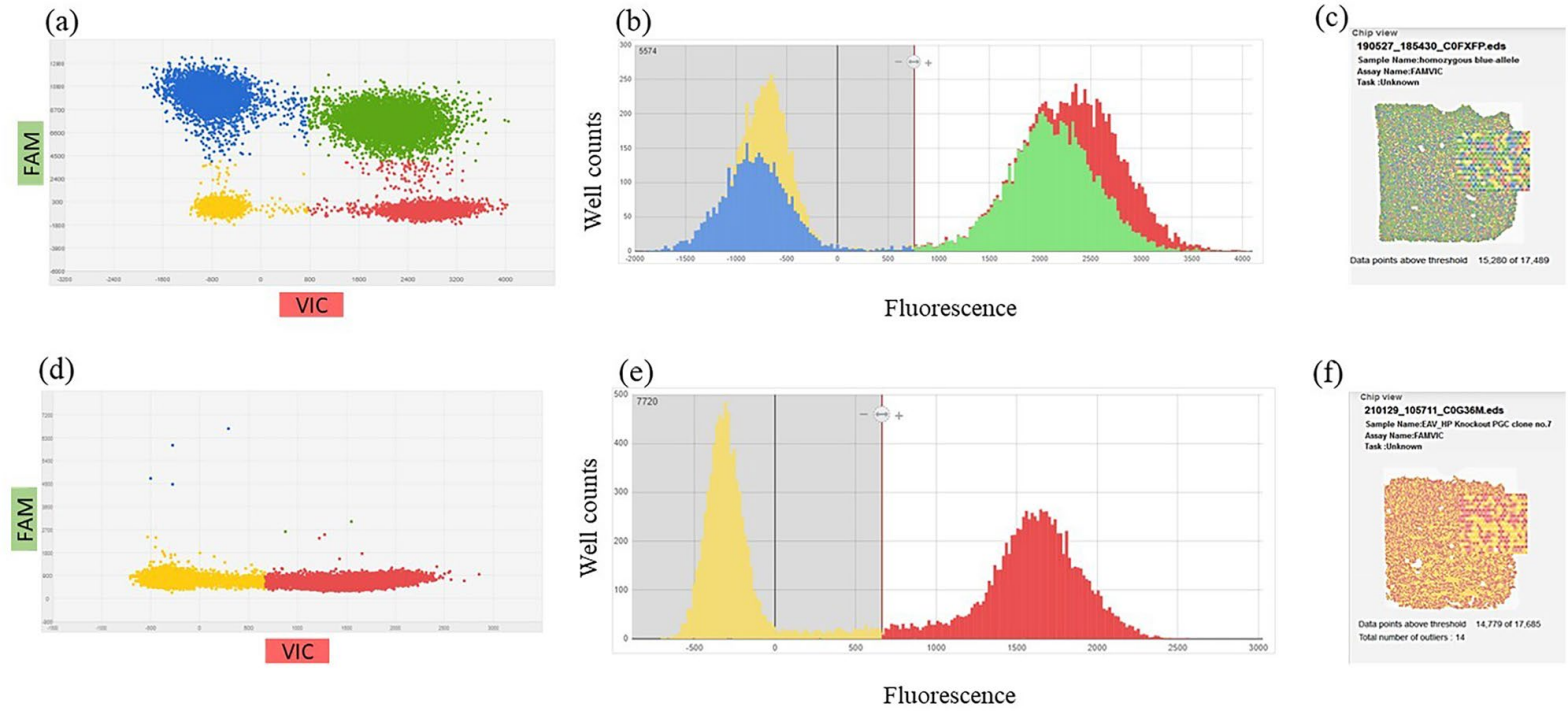
Digital PCR of homozygous blue-allele bearing PGCs and knock-out PGC clones (digital PCR 2). (**a,b,d,e**): blue: FAM reporter dye signal, red: VIC reporter dye signal, green: FAM/VIC reporter dye signals, yellow: no amplification (DNA-empty wells), VIC: reference assay (β-actin), FAM: digital PCR 2 assay. (**a**) Two-dimensional scatterplot of digital PCR duplex assay (FAM/VIC) and (**b**) histogram from PGCs (homozygous blue-allele bearing PGCs); (**c,f**) chip view by calls (uniformity), (**d**) scatterplot of digital PCR duplex assay (FAM/VIC) and (**e**) histogram from a clonal population of knock-out PGCs (homozygous deletion of the entire provirus insertion).

To substantiate the results, another digital PCR assay (digital PCR 2) was developed as a copy number variation assay (CNV) to compare the copy numbers of heterozygous and homozygous wildtype PGCs carrying the blue-allele with those of PGC knock-out clones. Again, the ß-actin assay (VIC) was set as biallelic reference. In this case, the second primer/probe combination resulted in an amplicon and fluorescence signal (FAM) only if the insertion was still present (Fig. S5). Figure 5 displays a comparison between the scatterplots and histograms of the homozygous blue-allele bearing PGCs and EAV-HP biallelic knock-out PGCs (Fig. 5a,b,d,e). For the knock-out PGCs no FAM signal was detected anymore. The chip view proved the good quality and uniformity of the reporter dye signals (Fig. 5c,f). Detailed digital PCR output data for the CNV assay analyzed with the QuantStudio 3D AnalysisSuite software are added as a supplementary Excel file. Table 2 lists the copy numbers of the different genotypes tested. Blue-allele bearing PGCs isolated from heterozygous and homozygous carrier chickens had almost half or identical copy numbers compared to the biallelic β-actin reference gene. Whereas five different knock-out PGC clones had similar copy number ratios to the nullizygous PGC control carrying no blue allele. The results confirmed that all five PGC cell clones tested exhibited a biallelic knock-out of the entire EAV-HP sequence.

**Table 2:** Results of digital PCR 2 of PGCs with different genotypes (HF-SpCas9).

Assay	Sample	Mean copies/µl
FAM	Homozygous blue-allele bearing PGCs	1562.2
VIC		1340.5
FAM	Heterozygous blue-allele bearing PGCs	395.0
VIC		752.3
FAM	Nullizygous blue-allele bearing PGCs	0.7
VIC		1758.8
FAM	EAV-HP knock-out PGCs (cell clone no.7) HF-SpCas9	0.5
VIC		1068.1
FAM	EAV-HP knock-out PGCs (cell clone no.5) HF-SpCas9	0.6
VIC		720.7
FAM	EAV-HP knock-out PGCs (cell clone no.3) HF-SpCas9	0.3
VIC		834.8
FAM	EAV-HP knock-out PGCs (cell clone no.4) HF-SpCas9	0.8
VIC		1082.5
FAM	EAV-HP knock-out PGCs (cell clone no.12) HF-SpCas9	0.5
VIC		907.3
FAM	No DNA control	0.4
VIC		0.07

To test the quantitative range of the digital PCR 2, we performed a serial dilution of DNA of homozygous blue-allele bearing PGCs in a constant background of chicken genomic DNA without binding site for the FAM-labeled probe (Fig. 4c). The observed copies per microliter (FAM) were plotted against the theoretical quantities (Fig. 4d). Simple linear regression analysis revealed high linearity with an R^2^ of 0.97. Quantification of FAM-positive partitions was detected in a range from 970 copies per microliter down to 2 copies per microliter at consistently high levels of background DNA.

In addition, the 69% of knock-out alleles in formerly homozygous blue-allele PGCs determined by digital PCR 1 were also determined by digital PCR 2, with 34% remaining wildtype alleles identified (Fig. S6).

### Characterization of knock-out clones

**Figure 6:**
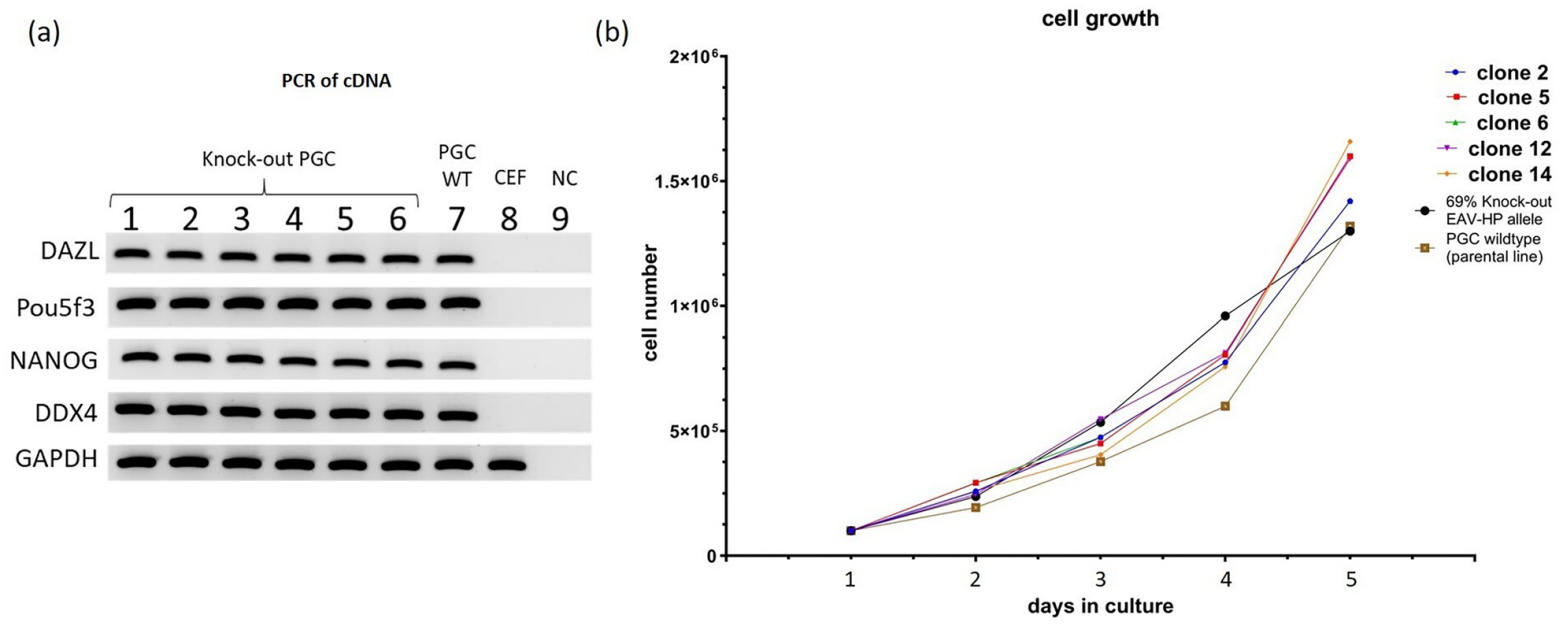
Committed stem cell character and growth curves of PGCs. (**a**) Marker gene expression of knock-out clones (1–6), wildtype PGCs (7) and chicken embryo fibroblasts (CEF) (8), 9: blank—no cDNA control (NC), pluripotency marker (PCR of cDNA): *NANOG, Pou5f3*, stem cell marker: *DAZL, DDX4*, housekeeping gene: *GAPDH,* 1–5: 5 representative knock-out cell clones (no. 2, 5, 6, 12 and 14, see Fig. S4), 6: knock-out clone (mixed cell population with 69% EAV-HP knock-out alleles (see Table 1), (**b**) PGC growth curves of wildtype and knock-out PGCs. The cell count was initially set at 100.000 cells and the cell count was determined over 5 days using a hemocytometer. The doubling time ranged from 30 to 32 h (Table S5). For uncropped versions of (**a**) see Fig. S12.

All established knock-out single-cell clones, as well as the wildtype PGCs and the PGCs with 69% altered alleles, were first cryopreserved and then thawed to confirm and compare the stem cell character and cell growth. We examined the expression of the PGC-specific genes *Pou5f3, NANOG, DAZL* and *DDX4* in PGCs and in chicken embryo fibroblasts (CEFs) (Fig. 6a). The pluripotency markers *Pou5f3* and *NANOG* and the specific PGC stem cell markers *DAZL* and *DDX4* were expressed in both wildtype and knock-out PGCs. No expression was found in CEFs, with the exception of the housekeeping gene *GAPDH*. The growth curves and corresponding doubling times of the PGCs were documented over 5 days. All PGCs had comparable growth capacities (Fig. 6b) and viability between 90 and 95% (Table S5). Thus the knock-out PGC clones had comparable characteristics as the germline colonizing PGC described before^39^.

### Off-target analysis

The algorithms of CRISPOR (http://crispor.org) found no off-target sites with 100% identity to the gRNA (G1 and G2) sequences (Table S1). However, the CRISPOR algorithms predicted 84 and 69 off-targets with two or more deviations from the target sequence for G1 and G2, respectively. We designed primer pairs for three most putative off-target binding sites of each gRNA (Table S2). No off-target events with high editing efficiency were detected in any of the sequenced amplicons (Fig. S7).

## Discussion

Newly established long-term in vitro cultivation techniques for PGCs without loss of germline competence open the way for in vitro gene editing approaches^16,17^. The main advantage of in vitro over in vivo transfection of PGCs is the possibility to evaluate the efficiency of the used gene editing tool and to pre-select cells with the desired genetic modification before starting an animal experiment^40^. As we have demonstrated in this study, highly precise and seamless genetic modifications are possible using the CRISPR/Cas9 system, even for knock-out of large regions such as a provirus insertion (4.2 kb).

The use of two selected gRNAs flanking the EAV-HP insertion combined with two different Cas9 endonucleases, one wildtype and one high-fidelity variant, resulted in highly efficient elimination of the entire EAV-HP provirus sequence with 29% and 69% efficiency, respectively. In our case, using a high-fidelity variant of the Cas9 endonuclease resulted in approximately twice as many deleted alleles and, in addition, single-cell clones with seamless deletion of the provirus were found. Previous studies have demonstrated that high-fidelity Cas9 variants have higher on-target specificity, as targets that have incomplete complementarities or mismatches with the corresponding gRNA are less likely to be cleaved^41,42^. For this reason, the commonly used guanine (G) extension of the gRNA sequence, which acts as a transcription initiator for U6 promoter-based gRNA expression, results in lower on-target efficiency^34,43^. In our case, the ‘G’ was part of the genomic target sequence and we did not need to prefix it. Furthermore, off-target prediction by CRISPOR did not detect another 100% on-target elsewhere in the chicken genome^44^. Although Sanger sequencing of some of the potential off-target PCR amplicons in this study did not reveal high off-target activity, this method is not deep enough to find off-targets with low editing efficiency. To this end, NGS-based methods need to be used to determine all potential off-targets^45^.

In a previous study, different high-fidelity Cas9 endonucleases have been tested and ranked in terms of efficiency, but the order was different for every target site tested^42^. Overall, thoughtful gRNA design and testing of different gRNAs and Cas9 variants still seems to be essential to achieve the desired target outcome.

Measuring of genome editing outcomes can be a critical and labor-intensive process. The T7 endonuclease I assay is a suitable test to get a first impression of the targeting efficiency of the selected guide RNAs and associated Cas9 enzyme in transfected cells due to its relatively simple and fast performance^46,47^. Under optimal conditions, the T7 assay indicates a detection limit in the range of approximately 5% of edited cells within a mixed cell population, based on an evaluation of the band intensity on the agarose gel^46,48^. In our study, intense additional bands of expected size were visible on the agarose gel after T7E1 digestion of the heteroduplex PCR products, suggesting that we had high on-target efficiencies for both gRNAs, even though no exact quantification can be done with this method. The quantification of gene editing events by digital PCR provides accurate information with a detection limit of 0,02%^49^, with no standard curve requirements. Here we used digital PCR as a highly specific and quantitative method to evaluate the efficiency of the gene editing outcome in a mixed PGC population including also a larger proportion of non-edited wildtype alleles. With the digital PCR 1 assay of this study, low copy numbers cannot be interpreted accurately, but such low potential efficiencies (0.17%) are not of concern for performing single-cell dilution of transfected PGCs. These false positive counts might be due to fluorescence of foreign particles, target nucleotide contamination, non-specific amplification of the polymerase or primer-dimer formation^50^.

It has already been demonstrated that digital PCR is a simple way to predict the efficiency of NHEJ-induced point mutations^51^. These assays are typically designed as drop-off assays where probes labelled with different dyes (FAM or HEX) compete for the binding site at the same genomic location. Our data demonstrated that with an individual assay design, even large genomic deletions induced by the CRISPR/Cas9 system can be quantified in a duplex assay with one probe binding at a reference gene like β-actin (*ACTB*). This gene located on chicken chromosome 14 is well suited as a biallelic reference gene, as it is species-specific without any allelic variation and occurs as a single copy in the chicken genome^52^.

In our study, it was essential to generate single PGC clones by limited dilution to verify by digital PCR whether the provirus deletion occurs on one or both alleles in the former homozygous blue-allele bearing PGCs. We found that each tested cell clone population had biallelic targeted alleles. Deletion of the provirus in both alleles was the desired outcome, because heterozygous blue-allele carrying Araucana chicken would continue to lay blue eggs^30,31^. In other cases, monoallelic disruption is needed, such as when modeling specific diseases or when a biallelic knock-out is lethal. Idoko et al. (2018) demonstrated that combining the use of a high fidelity SpCas9-HF1 with oligonucleotide mediated HDR increased precise mono- and biallelic editing events in chicken primordial germ cells^41^. The use of duplex digital PCR assays is a precise and quantitative method to detect both mono- or biallelic editing events in cell clones by simply evaluating copy numbers^53,54^.

Other groups also demonstrated that digital PCR is a rapid, accurate and cost-effective method for screening for successful genome editing^49,51,53,55^, including large DNA excisions and inversions^56^, even though it does not provide sequencing-level data. Compared to digital PCR, NGS-based methods such as targeted deep sequencing offer the ability to simultaneously analyze on-target and potential off-target sites, but bioinformatics skills are required to analyze the data and it remains expensive, which might not be amenable to smaller labs and small-scale CRISPR studies^45^. The 4.2 kb EAV-HP provirus insertion, especially the envelope gene (env) and the long terminal repeats (LTRs) sequences of this provirus exist in the Gallus genus at approximately 10 to 15 copies per genome^57–59^. Using short-read sequencing, it would be difficult to perform reliable alignment of these repetitive sequences. Long-read sequencing methods such as PacBio's (Pacific Biosciences) single-molecule real-time (SMRT)^60^ or nanopore sequencing (Oxford Nanopore Technologies (ONT))^61^ would have to be used in this case.

With our results, we demonstrated that after testing multiple gRNAs, one CRISPR/Cas9 guide pairing successfully eliminated the entire provirus (EAV-HP) on chromosome 1 in male chicken cell lines carrying the blue allele. Digital PCR easily demonstrated the high efficiency of our protocols.

## Materials and methods

### Animal experiments

Lohmann Breeders GmbH (Cuxhaven, Germany) donated fertilized eggs from an intercross of a population that was heterozygous for the blue eggshell color locus. For our experiments, we used a homozygous blue-allele bearing cell line which was established from this intercross in previous work^39^. These eggs derived from a case study which was part of the EU-project Innovative Management of Animal Genetic resources (IMAGE).

### PGC derivation and culture conditions

For performing the CRISPR/Cas9 experiments one male blood-derived PGC line (homozygous blue-allele bearing) was used. This cell line was established as follows. Animals carrying the blue egg allele in heterozygous state on a White Leghorn background line were mated and fertile eggs were incubated for 65 h to obtain Hamburger and Hamilton stages (HH) 14–16.

The PGCs were derived from fertilized eggs, which carry the blue-allele in homozygous state, and cultured in suspension without a feeder-layer and sub-cultured as described^62^. The customized avian KO-DMEM (CaCl_2_-free, 12.0 mM glucose, 250 mOsm) produced by ThermoFisher Scientific was used as the basal medium. It was supplemented with 1× B-27 supplement (ThermoFisher Scientific), 2.0 mM GlutaMax (ThermoFisher Scientific), 1× NEAA (Sigma), 0.1 mM β-mercaptoethanol (ThermoFisher Scientific), 1 × nucleosides (Sigma), 0.4 mM pyruvate (ThermoFisher Scientific), 0.2% ovalbumin (Sigma), 0.1 mg/ml sodium heparin (Sigma), 0.15 mM calcium chloride (Roth), 12.5 ng/ml human activin A (PeproTech), 4 ng/ml basic fibroblast growth factor (PeproTech), and 0.2% chicken serum (ThermoFisher Scientific).

### gRNA design and cloning

The gRNAs were designed using CRISPOR (http://crispor.org). The EAV-HP sequence was taken from the NCBI databank (GenBank accession no: KC632578). The EAV-HP insertion site in Araucana chicken is located on Chr.1. Geneious software (Geneious version 2021.0 created by Biomatters. Available from https://www.geneious.com) was used to rebuild the insertion site (Fig. S8). The complete EAV-HP insertion site on chr. 1 is shown in Fig. S9. Parts of this arranged sequence were used as template for CRISPOR. Prior to gRNA design, a long-range PCR described by Wragg et al. (2013) was performed in order to verify the identity of the target sequence in the used PGC lines. Guide RNA Oligo sequences are listed in Table S1.

### Transfection of PGCs

1 × 10^6^ PGCs were pelleted, washed once with 5 ml PBS 1× (1200 rpm, 3 min) and suspended in 200 µl Opti-MEM with 10 µg plasmid-DNA (max. 10% of total reaction volume). Both plasmids (PX459-derivates) carry the *Streptococcus pyogenes* derived Cas9 endonuclease (wildtype or high-fidelity variant) and the gRNA sequence on a single plasmid. The electroporation protocol (1300 V, 10 ms, 4 pulse) was performed using the Neon Transfection System (ThermoFisher Scientific, 100 µl Kit). Puromycin selection (0.5 µg/ml) of the transfected PGCs was started 24 h after transfection for up to 3 days. In parallel, non-transfected PGCs were puromycin treated to assess the efficacy of the antibiotic supplementation.

### On-target efficiency of CRISPR/Cas9 application

The target sequence was amplified via PCR using target specific primers (Table S3) under the following conditions: 95 °C for 2 min, 94 °C for 45 s, annealing at 57–60 °C (see Table S3 for details) for 45 s, 72 °C for 45 s and a final extension of 72 °C for 5 min for 35 cycles (Promega GoTaq Polymerase). Final elongation was performed at 72 °C for 5 min. To assess on-target efficiency of the guide RNAs of the CRISPR/Cas9 system the unpurified PCR product was used for T7 endonuclease-I cleavage assay (NEB). After enzymatic cleavage the reaction products were resolved using a 1.5% ultrapure agarose (Invitrogen) gel electrophoresis run at 80 V for 45 min in 1× TBE-buffer, and visualized using a transilluminator. If additional gel bands were detected, the specific PCR product was purified and Sanger sequenced. For Sanger sequencing (LGC Genomics GmbH) the PCR product was purified using the Invisorb Fragment Cleanup Kit (Invitek Molecular). For generating single cell PCR clones, the PCR product was cloned using the pGEM-T Easy Vector system (Promega) by following supplier’s instructions.

### dPCR

All three assays (digital PCR 1, digital PCR 2 (CNV), β-actin) were designed with the IDT PrimerQuest Tool and ordered from IDT as a set of two primers (900 nm) and one hydrolysis probe (250 nm). The ß-actin assay (HEX dye-labelled) was used as reference assay (biallelic). Primer and probe sequences are listed in Table S4. The chip-based dPCR (QuantStudio 3D Digital PCR system, ThermoFisher Scientific) was performed in a total reaction volume of 14.5 μl as follows: 7.3 μl QuantStudio3D Digital PCR Master Mix v2 (ThermoFisher Scientific), 0.7 μl HEX and FAM dye-labeled assays each, 1.4 μl diluted genomic DNA, c = 25 ng/µl), and 4.4 μl nuclease-free water. Standard dPCR thermal cycling conditions recommended by the supplier were used with an annealing temperature of 60 °C (ProFlex 2× Flat PCR 93 System). End-point fluorescence data files, generated by the QuantStudio 3D Digital PCR Instrument, were analyzed using the QuantStudio 3D AnalysisSuite software (Excel files, see Supplements). The quantification algorithm Poisson Plus was set with a confidence level of 95% and a desired precision of 10% (default value). All three digital PCR assays used (ß-actin, digital PCR 1 FAM target, and digital PCR 2 FAM target) were tested individually, and the results are shown in Fig. S10. Four serial dilution steps (dPCR1: 1:2, 1:3, 1:10, 1:10; dPCR2:1:3, 1:2; 1:10, 1:10) were performed with DNA from a knock-out cell clone (dPCR1) and DNA from homozygous blue-allele bearing PGCs (dPCR2). Each dilution step was performed in chicken genomic DNA that did not have a binding site for the FAM-labeled probe, but only for the VIC probe. Each dilution step was quantified in duplicates, but due to losses of DNA during sample preparation and chip loading, less starting DNA was obtained for the duplicate. Digital PCR was used to quantify the exact initial concentration because UV-spectrophotometry overestimates the DNA concentration. R^2^ values were calculated using simple linear regression analysis in GraphPad Prism version 9.0.0 (121) for Windows, GraphPad Software, San Diego, California USA, https://www.graphpad.com.

### RNA isolation and cDNA synthesis

Total RNA was isolated from pelleted PGCs (1 × 10^6^) using 1 ml TRIzol Reagent (ThermoFisher scientific) and phenol–chloroform extraction as described^63^. For cDNA synthesis the High-Capacity cDNA Reverse Transcription Kit (Applied Biosystem), including random primer, was used according to the manufacturer’s guidelines. 1 µg RNA was used for reverse transcription.

### Reverse transcription PCR

PCR conditions were 95 °C for 2 min, 94 °C for 45 s, 60 °C for 45 s, 72 °C for 45 s and a final extension of 72 °C for 5 min for 34 cycles (Promega GoTaq Polymerase). Reaction products were resolved using a 1.5% ultrapure agarose (Invitrogen) gel electrophoresis run at 80 V for 45 min in 1× TBE-buffer, and visualized using a transilluminator. Intron-spanning primer were used as in our previous studies^39^.

### Off-target analysis

For each gRNA, three different primer pairs were designed to amplify the three highest ranked off-targets sites predicted by CRISPOR^44^. Primer sequences and specific annealing temperatures are listed in Table S2. PCR conditions were as described for on-target analysis (Promega GoTaq Polymerase). The reaction products were purified and Sanger sequenced.

### Ethics declarations

Authors have no conflict of interest to declare.

## Supplementary Information

Supplementary Information 1.

Supplementary Information 2.

## Data Availability

The data supporting the findings of the present study are available from the corresponding author upon reasonable request.

## References

[1] Schuster F (2020). CRISPR/Cas12a mediated knock-in of the polled Celtic variant to produce a polled genotype in dairy cattle. Sci. Rep..

[2] Kurtz S (2021). Knockout of the HMG domain of the porcine SRY gene causes sex reversal in gene-edited pigs. Proc. Natl. Acad. Sci. USA.

[3] Hein R (2020). Triple (GGTA1, CMAH, B2M) modified pigs expressing an SLA class I(low) phenotype—Effects on immune status and susceptibility to human immune responses. Am. J. Transplant..

[4] Bosch P (2015). Exogenous enzymes upgrade transgenesis and genetic engineering of farm animals. Cell Mol. Life Sci..

[5] McFarlane GR, Salvesen HA, Sternberg A, Lillico SG (2019). On-farm livestock genome editing using cutting edge reproductive technologies. Front. Sustain. Food Syst..

[6] Perisse IV, Fan Z, Singina GN, White KL, Polejaeva IA (2020). Improvements in gene editing technology boost its applications in livestock. Front. Genet..

[7] Kalds P (2019). Sheep and goat genome engineering: From random transgenesis to the CRISPR Era. Front. Genet..

[8] Woodcock ME, Idoko-Akoh A, McGrew MJ (2017). Gene editing in birds takes flight. Mamm. Genome.

[9] Sid H, Schusser B (2018). Applications of gene editing in chickens: A new era is on the horizon. Front. Genet..

[10] Rieblinger B (2021). Cas9-expressing chickens and pigs as resources for genome editing in livestock. Proc. Natl. Acad. Sci. USA.

[11] Hellmich R (2020). Acquiring resistance against a retroviral infection via CRISPR/Cas9 targeted genome editing in a commercial chicken line. Front. Genome Ed..

[12] Oishi I, Yoshii K, Miyahara D, Tagami T (2018). Efficient production of human interferon beta in the white of eggs from ovalbumin gene-targeted hens. Sci. Rep..

[13] Ballantyne M (2021). Direct allele introgression into pure chicken breeds using Sire Dam surrogate (SDS) mating. Nat. Commun..

[14] Kim GD (2020). Generation of myostatin-knockout chickens mediated by D10A-Cas9 nickase. FASEB J..

[15] Bellairs R, Osmond M (2005). The Atlas of Chick Development.

[16] Whyte J (2015). FGF, Insulin, and SMAD signaling cooperate for avian primordial germ cell self-renewal. Stem Cell Rep..

[17] van de Lavoir MC (2006). Germline transmission of genetically modified primordial germ cells. Nature.

[18] Panda SK, McGrew MJ (2021). Genome editing of avian species: implications for animal use and welfare. Lab Anim..

[19] Petersen B, Niemann H (2015). Molecular scissors and their application in genetically modified farm animals. Transgenic Res..

[20] Gaj T, Gersbach CA, Barbas CF (2013). ZFN, TALEN, and CRISPR/Cas-based methods for genome engineering. Trends Biotechnol..

[21] Danner E (2017). Control of gene editing by manipulation of DNA repair mechanisms. Mamm. Genome.

[22] Ran FA (2013). Genome engineering using the CRISPR-Cas9 system. Nat. Protoc..

[23] Jinek M (2013). RNA-programmed genome editing in human cells. Elife.

[24] Oishi I, Yoshii K, Miyahara D, Kagami H, Tagami T (2016). Targeted mutagenesis in chicken using CRISPR/Cas9 system. Sci. Rep..

[25] Park TS, Park J, Lee JH, Park JW, Park BC (2019). Disruption of G0/G1 switch gene 2 (G0S2) reduced abdominal fat deposition and altered fatty acid composition in chicken. FASEB J..

[26] Koslova A (2020). Precise CRISPR/Cas9 editing of the NHE1 gene renders chickens resistant to the J subgroup of avian leukosis virus. Proc. Natl. Acad. Sci. USA.

[27] Ihry RJ (2018). p53 inhibits CRISPR-Cas9 engineering in human pluripotent stem cells. Nat. Med..

[28] Bloom JC, Loehr AR, Schimenti JC, Weiss RS (2019). Germline genome protection: Implications for gamete quality and germ cell tumorigenesis. Andrology.

[29] Baarends W, Laan RVD, Grootegoed J (2001). DNA repair mechanisms and gametogenesis. Reproduction.

[30] Wragg D (2013). Endogenous retrovirus EAV-HP linked to blue egg phenotype in Mapuche fowl. PLoS ONE.

[31] Wang Z (2013). An EAV-HP insertion in 5' Flanking region of *SLCO1B3* causes blue eggshell in the chicken. PLoS Genet..

[32] Li Z (2019). Association between the methylation statuses at CpG sites in the promoter region of the SLCO1B3, RNA expression and color change in blue eggshells in Lushi chickens. Front. Genet..

[33] Kato-Inui T, Takahashi G, Hsu S, Miyaoka Y (2018). Clustered regularly interspaced short palindromic repeats (CRISPR)/CRISPR-associated protein 9 with improved proof-reading enhances homology-directed repair. Nucleic Acids Res..

[34] Kleinstiver BP (2016). High-fidelity CRISPR-Cas9 nucleases with no detectable genome-wide off-target effects. Nature.

[35] Sternberg SH, LaFrance B, Kaplan M, Doudna JA (2015). Conformational control of DNA target cleavage by CRISPR–Cas9. Nature.

[36] Knight SC (2015). Dynamics of CRISPR-Cas9 genome interrogation in living cells. Science.

[37] Majumdar N, Wessel T, Marks J (2015). Digital PCR modeling for maximal sensitivity, dynamic range and measurement precision. PLoS ONE.

[38] Demeke T, Dobnik D (2018). Critical assessment of digital PCR for the detection and quantification of genetically modified organisms. Anal. Bioanal. Chem..

[39] Altgilbers S, Klein S, Dierks C, Weigend S, Kues WA (2021). Cultivation and characterization of primordial germ cells from blue layer hybrids (*Araucana* crossbreeds) and generation of germline chimeric chickens. Sci. Rep..

[40] Cooper CA, Doran TJ, Challagulla A, Tizard MLV, Jenkins KA (2018). Innovative approaches to genome editing in avian species. J. Anim. Sci. Biotechnol..

[41] Idoko-Akoh A, Taylor L, Sang HM, McGrew MJ (2018). High fidelity CRISPR/Cas9 increases precise monoallelic and biallelic editing events in primordial germ cells. Sci. Rep..

[42] Kulcsar PI (2017). Crossing enhanced and high fidelity SpCas9 nucleases to optimize specificity and cleavage. Genome Biol..

[43] Zhang D (2017). Perfectly matched 20-nucleotide guide RNA sequences enable robust genome editing using high-fidelity SpCas9 nucleases. Genome Biol..

[44] Haeussler M (2016). Evaluation of off-target and on-target scoring algorithms and integration into the guide RNA selection tool CRISPOR. Genome Biol..

[45] Bennett EP (2020). INDEL detection, the 'Achilles heel' of precise genome editing: A survey of methods for accurate profiling of gene editing induced indels. Nucleic Acids Res..

[46] Vouillot L, Thelie A, Pollet N (2015). Comparison of T7E1 and surveyor mismatch cleavage assays to detect mutations triggered by engineered nucleases. G3 (Bethesda).

[47] Zischewski J, Fischer R, Bortesi L (2017). Detection of on-target and off-target mutations generated by CRISPR/Cas9 and other sequence-specific nucleases. Biotechnol. Adv..

[48] Kim Y (2013). A library of TAL effector nucleases spanning the human genome. Nat. Biotechnol..

[49] Findlay SD, Vincent KM, Berman JR, Postovit LM (2016). A digital PCR-based method for efficient and highly specific screening of genome edited cells. PLoS ONE.

[50] Hunter ME (2017). Detection limits of quantitative and digital PCR assays and their influence in presence–absence surveys of environmental DNA. Mol. Ecol. Resour..

[51] Miyaoka, Y., Mayerl, S. J., Chan, A. H. & Conklin, B. R. *Digital PCR Methods in Molecular Biology*. Chap. 20. 349–362 (2018). 10.1007/978-1-4939-7778-9_20 PMC6372302 29717453

[52] Xiang W (2017). Identification of a chicken (*Gallus gallus*) endogenous reference gene (Actb) and its application in meat adulteration. Food Chem..

[53] Mock U, Hauber I, Fehse B (2016). Digital PCR to assess gene-editing frequencies (GEF-dPCR) mediated by designer nucleases. Nat. Protoc..

[54] Peng C (2020). Accurate detection and evaluation of the gene-editing frequency in plants using droplet digital PCR. Front. Plant Sci..

[55] Sedlak RH (2016). Digital detection of endonuclease mediated gene disruption in the HIV provirus. Sci. Rep..

[56] Watry HL (2020). Rapid, precise quantification of large DNA excisions and inversions by ddPCR. Sci. Rep..

[57] Sacco MA, Flannery DM, Howes K, Venugopal K (2000). Avian endogenous retrovirus EAV-HP shares regions of identity with avian leukosis virus subgroup J and the avian retrotransposon ART-CH. J. Virol..

[58] Sacco MA, Howes K, Venugopal K (2001). Intact EAV-HP endogenous retrovirus in Sonnerat's jungle fowl. J. Virol..

[59] Sacco MA, Howes K, Smith LP, Nair VK (2004). Assessing the roles of endogenous retrovirus EAV-HP in avian leukosis virus subgroup J emergence and tolerance. J. Virol..

[60] Eid J (2009). Real-time DNA sequencing from single polymerase molecules. Science.

[61] Clarke J (2009). Continuous base identification for single-molecule nanopore DNA sequencing. Nat. Nanotechnol..

[62] Collarini EJ, Leighton PA, Van de Lavoir MC (1874). Production of transgenic chickens using cultured primordial germ cells and gonocytes. Methods Mol. Biol..

[63] Toni LS (2018). Optimization of phenol-chloroform RNA extraction. MethodsX.

