# Supplementary material for: Quantitative analysis of CRISPR/Cas9-mediated provirus deletion in blue egg layer chicken PGCs by digital PCR
Source: Sci Rep. 2022 Sep 16;12:15587. doi: 10.1038/s41598-022-19861-7 (PMC9481566; doi:10.1038/s41598-022-19861-7)
Supplement: Supplementary file 2 — Supplementary Information 2. [file 41598_2022_19861_MOESM2_ESM.pdf]

Supplementary information

Quantitative Analysis of CRISPR/Cas9-mediated Provirus Deletion in Blue Egg Layer Chicken PGCs by Digital PCR

Stefanie Altgilbers[1], Claudia Dierks[2], Sabine Klein[1], Steffen Weigend[2], Wilfried A. Kues[1]

Friedrich-Loeffler-Institut, Institute of Farm Animal Genetics,
Dept. Biotechnology, Stem Cell Physiology[1] and Dept. of Breeding and Genetic Resources[2],
31535 Neustadt, Germany

Tab. S1 CRISPR/Cas9 guide RNA oligonucleotide sequences

Successful guide RNAs	Forward	Reverse
G1	5'-CACCGGAGACTACATGCAACATGA	5'-AAACTCATGTTGCATGTAGTCTCC
G2	5'-CACCGCATACAACACCTTCAACAG	5'-AAACCTGTTGAAGGTGGTGTATGC
Unsuccessful guide RNA combinations (for proviral deletion)	Upstream chr.1	Downstream chr.1
1*	5'-CACCGCTAAATAAGCTTGGGAAAG	5'-CACCGCATACAACACCTTCAACAG
2*	5'-CACCGCGTAGATAAACATGTATTT	5'-CACCGCATACAACACCTTCAACAG
3*	5'-CACCGCTAAATAAGCTTGGGAAAG	5'-CACCGCATACAACACCTTCAACAG
4*	5'-CACCGCGTAGATAAACATGTATTT	5'-CACCGCCAGTTCTGCCCTCTGTTGA
5*	5'-CACCGCTAAATAAGCTTGGGAAAG	5'-CACCGCCAGTTCTGCCCTCTGTTGA
6*	5'-CACCGCGTAGATAAACATGTATTT	5'-CACCGCATACAACACCTTCAACAG
7*	5'-CACCGTACGCTGGTCAAAAATTAAT	5'-CACCGCTCCTTAAGCAAGACTGTGT

Overhangs

The gRNAs were tested in single transfection and co-transfection experiments. Evaluation of these testing was first performed using Standard PCR followed by T7 assay to demonstrate a workflow that was as simple and inexpensive as possible. No positive T7 assay result was observed using these gRNAs*, even in combination with each other. Sanger sequencing also confirmed that efficient editing was not present. Because the T7 assay has a detection limit of about 5%, we decided that the gRNAs that did not complete this assay positively were not worth testing further because the efficiency of genome editing would be too low to perform the labor-intensive single-cell dilution.

Tab. S2 Off-target gRNA and primer sequences for G1 and G2

Off-target (gRNA)	Off-target gRNA sequence	Genomic location (Off-target gRNA)[1]	Forward primer	Reverse primer	Amplicon (bp)	Annealing temperature (°C)
chr.5, *DPH6*, intronic (G1)	5'-GGAGACCACATGCAACAAGA	NC_052577.1:g.31504759-31504781	5'-TCTGGACCTTTAGAGTGTAAGTGT	5'-TGTGCCAAGTGTAGCTTCGT	409	60
chr.4, *EMCN*, intronic (G1)	5'-GGAGAAAATATGCAACATGA	NC_052576.1:g.59701205-59701224	5'-AGTTGCAAGTGGGAAGACGT	5'-AAGAAGCTGGCAGTTCACGT	474	60
chr.31, *LOC121112681*, intronic (G1)	5'-GGACACTGCATGCAGAATGA	NW_024096188.1:g.5719-5738	5'-TCACACAGCATCCCCAACTC	5'-AGAGCTGACGCAAAGAGACC	425	60
chr.18, *PITPNC1*, intronic (G2)	5'-ATATGCAACACCTTCAACAG	NC_052590.1:g.7326029-7326051	5'-ACTGGAACTTTAAAAGCCTTGCC	5'-ACTCTGTGCTGACTTGTACCC	442	60
chr.1, intergenic (G2)	5'-ACATACAATGCCTTCAACAG	NC_052573.1:g134913968-134913987	5'-TGTGGGAAAACTGGAGCTCC	5'-TCCAAGAAGCCGGCTCATTT	487	57
chr 1, *UTP20*, intronic (G2)	5'-GGCTCCAACAACTTCAACAG	NC_052573.1:g.48026532-48026551	5'-GCTGCCCTTTGACCAACAAG	5'-ATGCTGACCACGAGAGATGC	477	60

[1]assembly: GCA_016700215.2

Tab. S3 Primer sequences for amplifying CRISPR/Cas9 target regions

Primer	Forward	Reverse	Amplicon (bp)	Annealing temperature (°C)
1F/1R	5´-TGGCTCTATCAGTCTTGGATG-3´	5´-ACGCATACAACATCTCCCC-3´	595	58
2F/2R	5´-GGTCACCACTTGTTGCATG-3´	5´-GCGATTGGATTAACACTGC-3´	840	57
1F/2R	5´-TGGCTCTATCAGTCTTGGATG-3´	5´-GCGATTGGATTAACACTGC-3´	786 (deleted and wildtype allele)<br><br>5028 (Araucana allele, not amplified under the used conditions)	60

Tab. S4 Primer/Probe sequences for digital PCR

dPCR Assay	Forward	Probe	Reverse
digital PCR 1 EAV-HP	5´-TGGTACTTGGTAGAGGAATCATTAAC -3´	5´-/56-FAM/ACGGGTGTA/ZEN/CAAATAGAGCACACTTGCA/3IABkFQ/- 3´	5´-GCTCGGGAAAGCAGGCA- 3´
digital PCR 2 EAV-HP	5´-CGTCACACCTCGTTTCC-3´	5´-/56-FAM/CAA CAC ACC /ZEN/TCG TTT CCC TCG CTA /3IABkFQ/- 3´	5´AAGACTGTGTTGGTACTTGG- 3´
ß-Actin	5´-CCTCTTCCAGCCATCTTTC- 3´	5´-/5HEX/ACTCCTTTG/ZEN/AGGAGATTCCATGCCC/3IABkFQ/- 3´	5´-AATATTGTGGCCAGTCTCTAAG-3´

Araucana specific SNP; (HEX ≙VIC (almost identical Ex/Em)

Table S5 Cell counts and doubling time of PGCs

Day in culture	PGC cell clone						
	Knock-out single cell clone 2	Knock-out single cell clone 5	Knock-out single cell clone 6	Knock-out single cell clone 12	Knock-out single cell clone 14	Knock-out clone (69 % mutated EAV-HP alleles)	Wildtype PGCs
1	100.000	100.000	100.000	100.000	100.000	100.000	100.000
2	259.000	292.500	292.000	246.250	260.000	237.500	193.000
3	475.000	450.000	475.000	547.500	405.000	535.000	377.500
4	775.000	805.000	775.000	812.500	757.500	960.000	599.500
5	1.420.000	1.600.000	1.420.000	1.590.000	1.659.000	1.300.000	1.320.000
Doubling time (h)	31	30	31	30	30	32	32
Average cell viability %	93,6	91,3	90,6	95,2	93,3	93,6	92,3

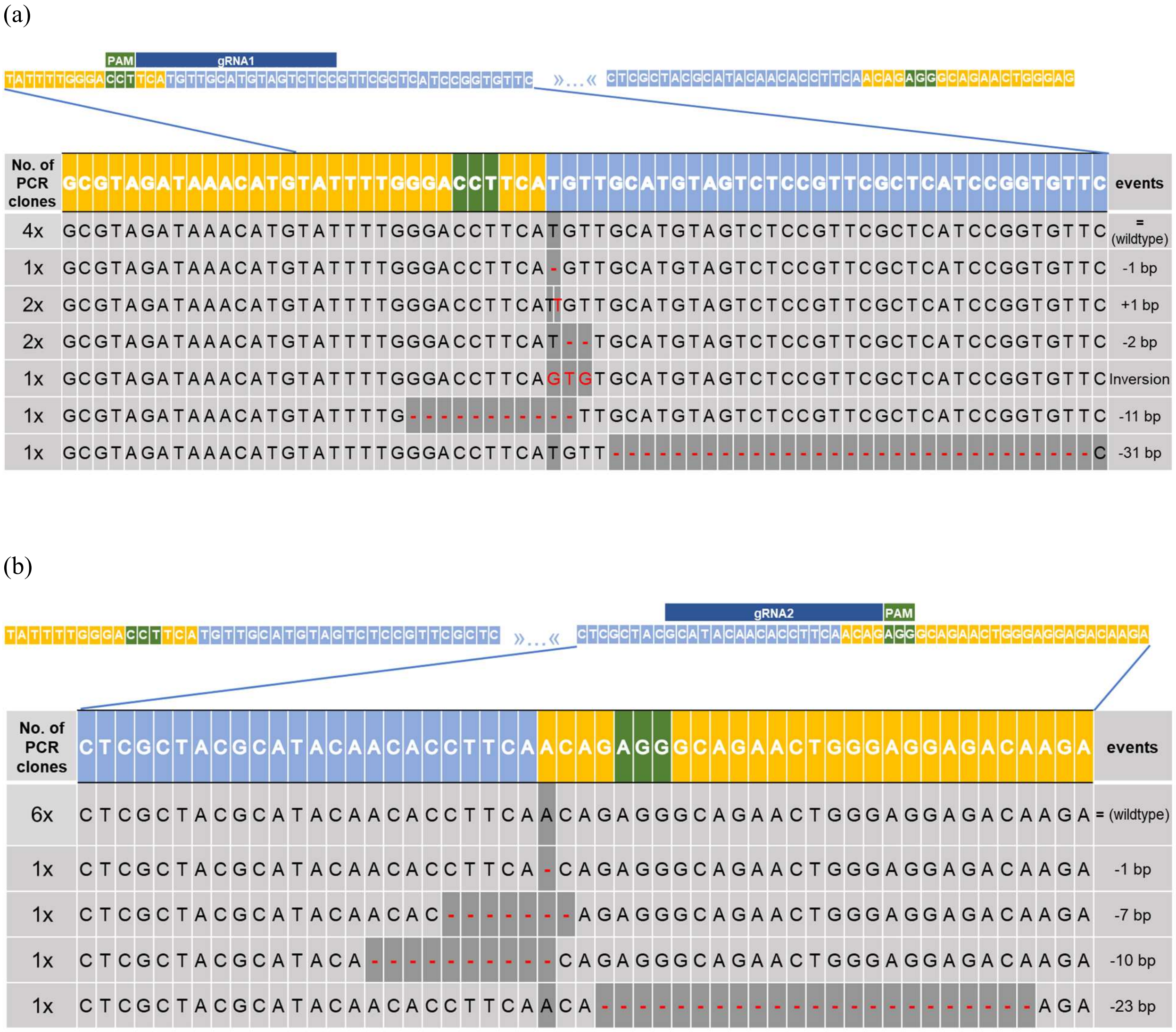

Fig. S1 CRISPR/Cas9-mediated Indels at the blue egg locus in Araucana crossbreed chicken PGCs after single transfection with gRNA1 or gRNA2 (high-fidelity SpCas9)

Yellow: Chromosome 1, light blue: EAV-HP retrovirus sequence, PAM: protospacer adjacent motif (green), dark blue: gRNAs; Sanger sequences were aligned to the insertion site of the EAV-HP sequence on chromosome 1 in Araucana crossbreed chicken.

(a) Sanger sequencing of 12 single PCR clones (1F/1R) based on single transfection of homozygous blue allele bearing PGCs with gRNA1; (b) Sanger sequencing of 10 single PCR clones (2F/2R) based on single transfection of homozygous blue allele bearing PGCs with gRNA2.

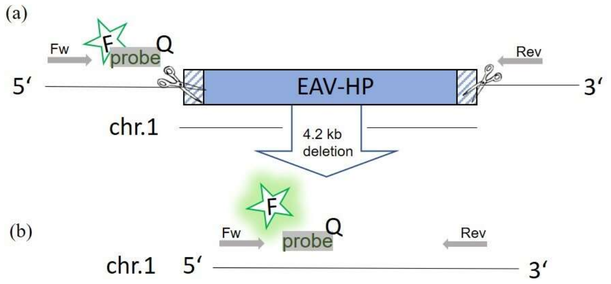

Fig. S2 Schematic diagram of the digital PCR assay design (digital PCR 1)

F = fluorophore; Q = quencher; Fw = forward primer; Rev = reverse primer

(a) Relative positions of primers and the hydrolysis probe to the provirus insertion (EAV-HP);

(b) A CRISPR/Cas9-mediated deletion of the provirus sequence leads to the amplification of the shortened mutated sequence, resulting in a detectable fluorescence signal.

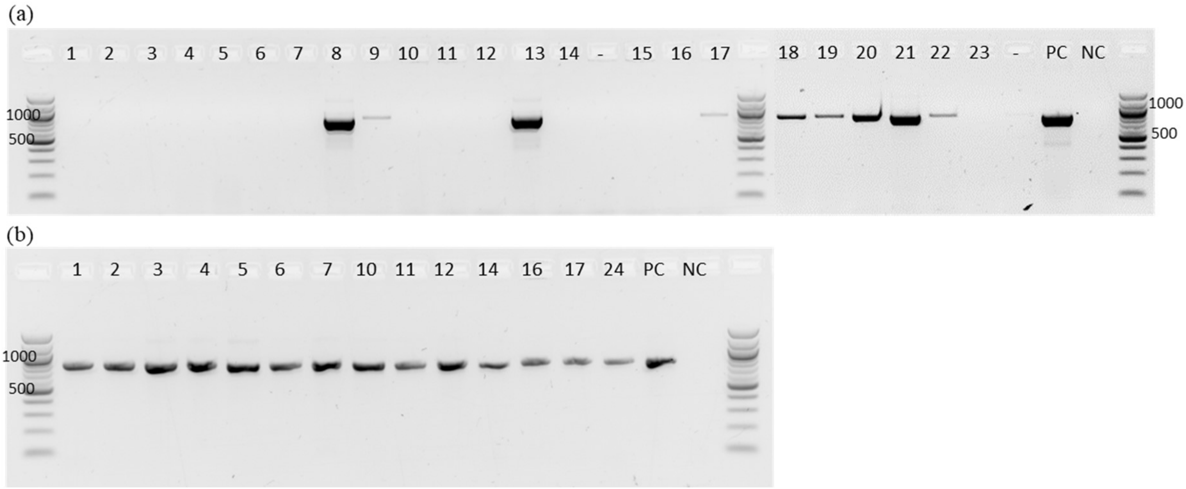

Fig. S3 PCR Genotyping of knock-out PGC single cell clones (high-fidelity SpCas9)

PC = positive control, NC = negative control, 100 bp ladder

(a) PCR 2F/2R: 9 PGC cell clones were the EAV-HP insertion is still present (8, 9, 13, 17, 18-22) and 14 PGC cell clones with no amplicon,

(b) The 14 knock-out single cell clones from (a) showed an amplicon using PCR 1F/2R (786 bp), confirming deletion of the EAV-HP sequence on chromosome 1 in the tested PGC cell clones

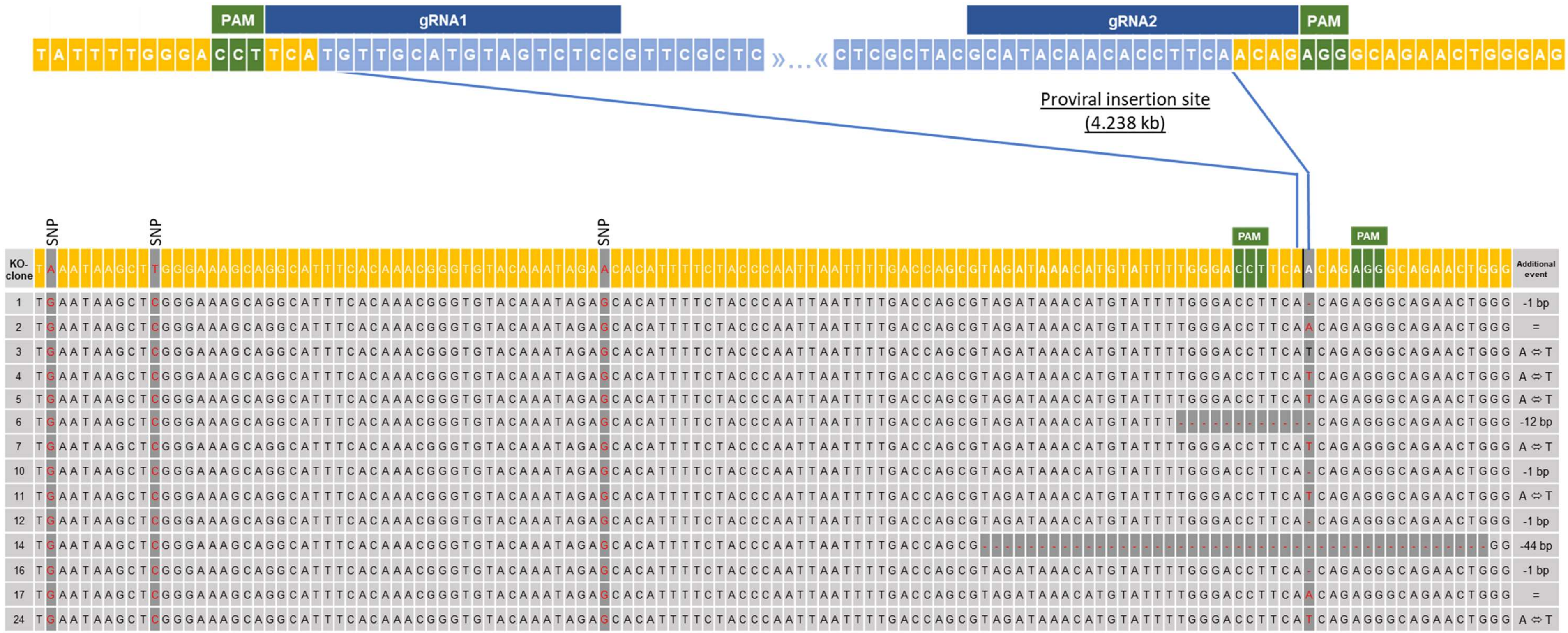

Fig. S4 Sanger sequencing of 14 PGC cell clones after knock-out of the entire EAV-HP provirus sequence (high-fidelity SpCas9)

The 14 tested clones were Sanger sequenced and aligned to the corresponding Sanger sequence of a non-blue allele bearing White Leghorn chicken. Araucana specific SNPs (1: A>G, 2: T>C, 3: A>G).

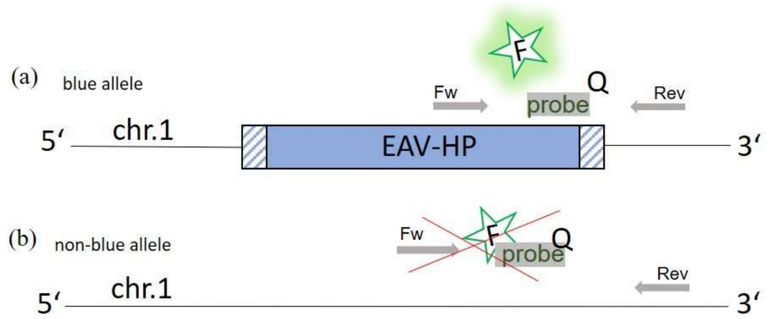

Fig. S5 Schematic diagram of the digital PCR assay design (digital PCR 2)
F = fluorophore; Q = quencher; Fw = forward primer; Rev = reverse primer
(a) Locations of primers and the hydrolysis probe in/near the provirus insertion (EAV-HP). The forward primer and probe bind to the provirus sequence (blue allele), and a fluorescent signal is detected;
(b) There is no amplification in case of a non-blue allele.

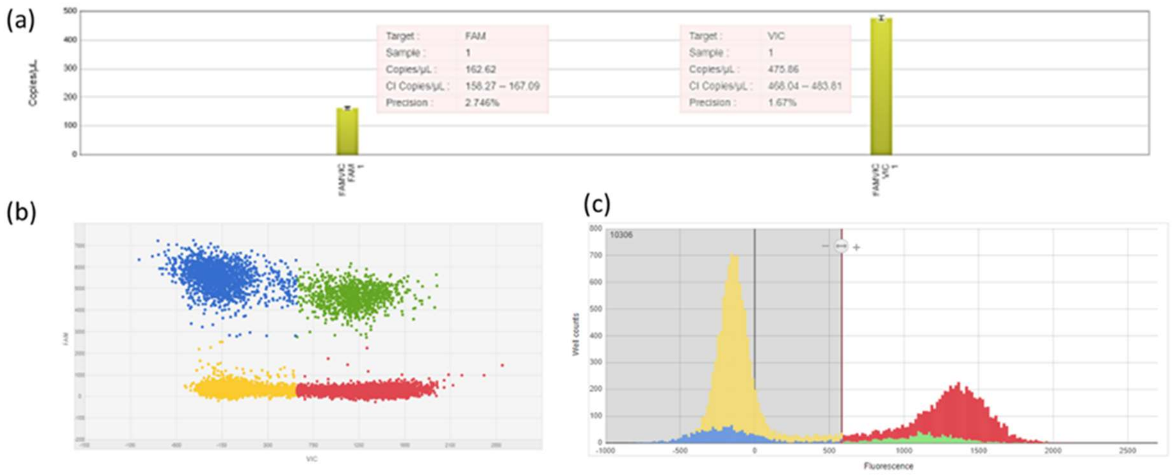

Fig. S6 Digital PCR of mixed knock-out cell population in dPCR2
(a) Column diagram of dPCR2 results (3 replicates), FAM = 162,62 copies (34 %), VIC = 475,86. (b) Scatterplot of dPCR2 (duplex assay) of mixed knock-out cell population (69 % EAV-HP knock-out alleles determined by dPCR1), (c) corresponding histogram to (a).

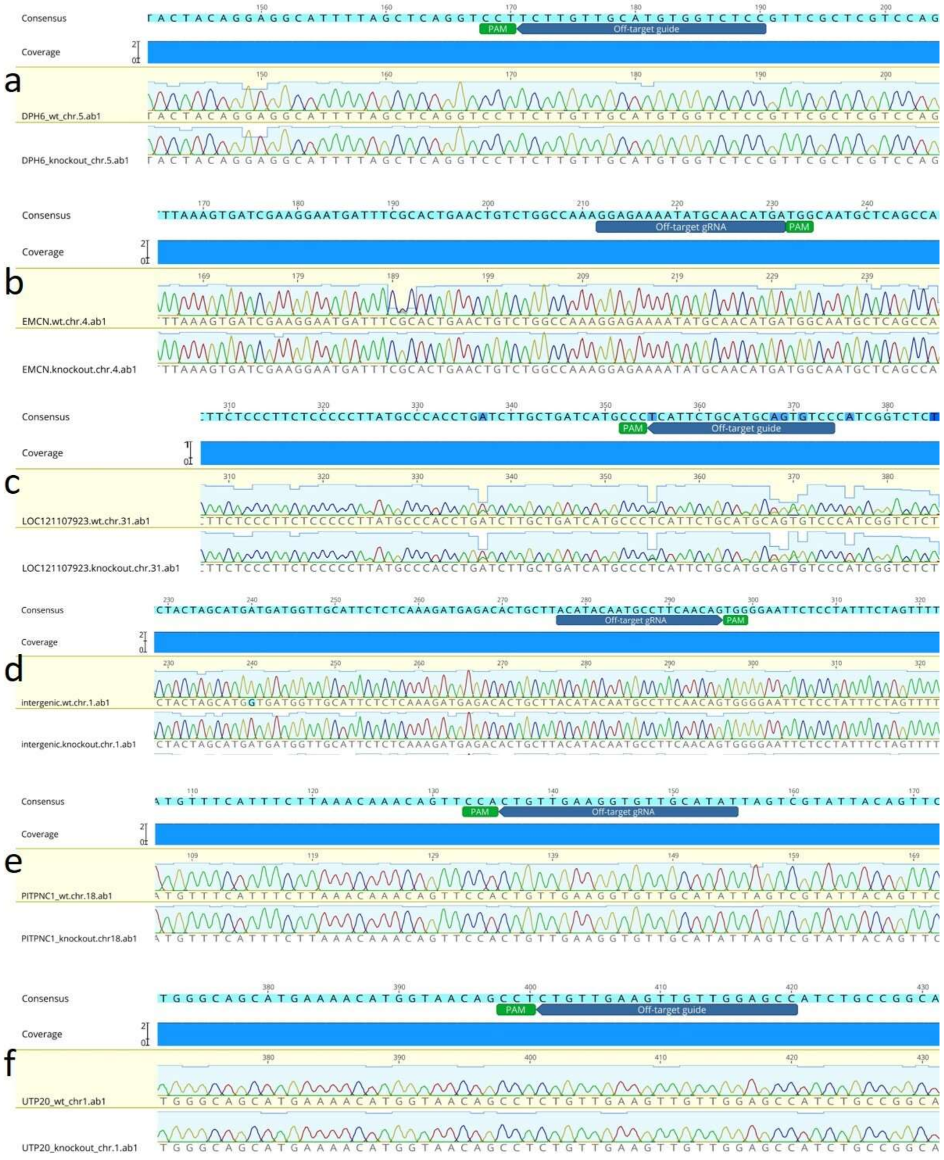

Fig. S7 Off-target analysis of G1 and G2 (Sanger sequencing for detecting potential off-target sites)

a-c = electropherograms of potential Off-target sequences of gRNA1 (G1), d-f= electropherograms of potential Off-target sequences of gRNA2 (G2), 1 = wildtype sequence (homozygous blue allele bearing PGC cell line, non-edited), 2 = knock-out PGC clone, dark blue = Off-target gRNA sequence, green = PAM; genomic location based on chicken genome assembly: GCA_016700215.2 (see Tab. S2)

(a) *DPH6*, chr.5, intronic; (b) *EMCN*, chr.4, intronic; (c) *LOC121112681*, chr.31, intronic; (d) intergenic, chr.1;

(e) *PITPNC1*, chr.18, intronic; (f) *UTP20*, chr.1, intronic; No Off-targets were found for the six tested Off-target positions (a-f).

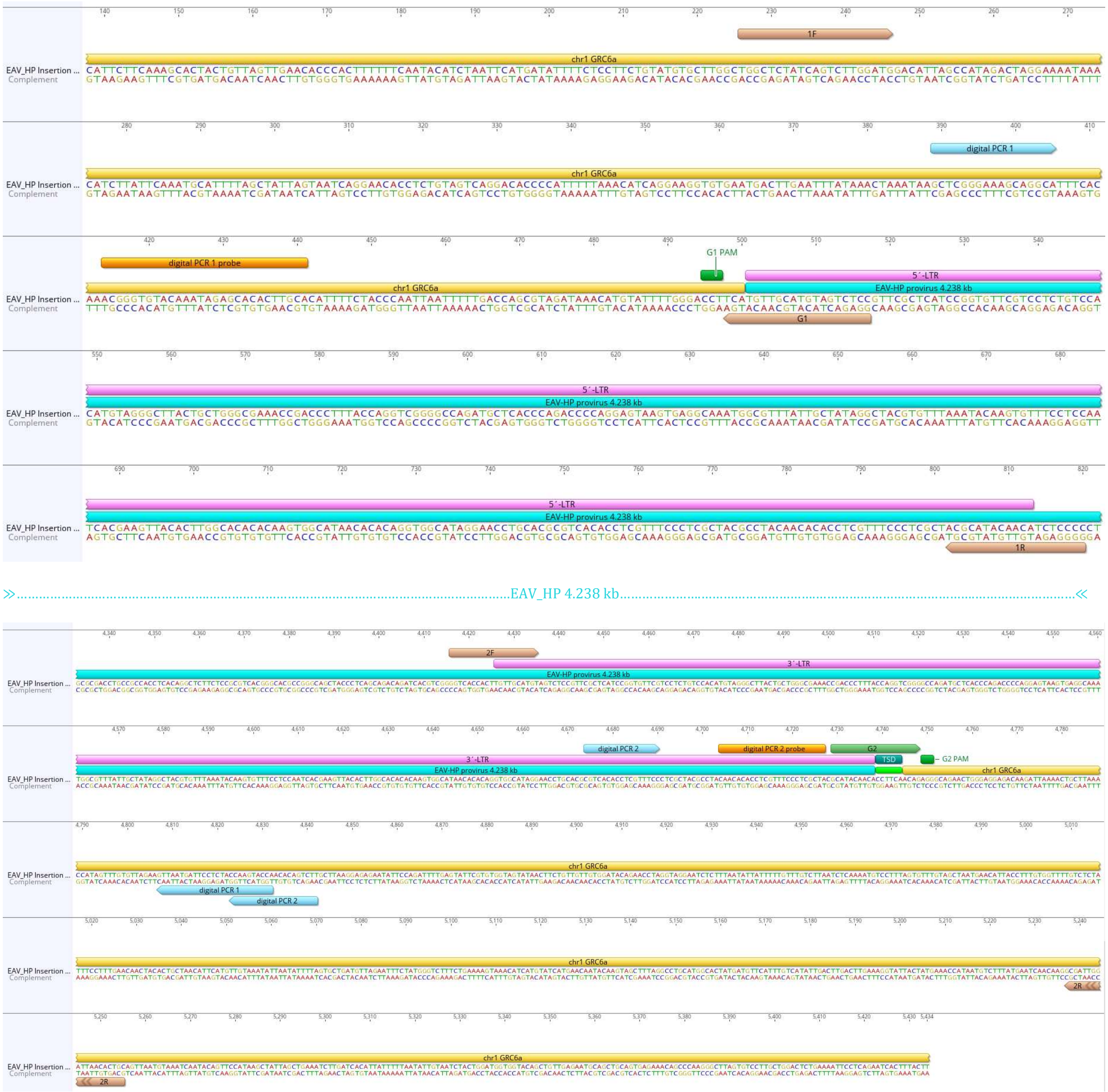

Fig. S8 EAV-HP provirus sequence on Araucana chicken chromosome 1 supplemented with assay specific annotations generated using Geneious software (*Geneious version 2021.0 created by Biomatters*)

brown: Primer for standard PCR (1F/1R; 2F/2R); pink: LTR = long terminal repeat sequence; TSD = target-site duplication; PAM = protospacer adjacent motif; G1 = gRNA1; G2 = gRNA2; orange: probes used in digital PCR (digital PCR1 & 2); light blue: primer used in digital PCR

GTTAGGAACCCAGTATGGGATTCCTTGTACTAGATCTGTAAGAAGGCAGAGGGTTGATCCAAAGTATACATAGCCCCAATATACTTTCAGGTACGCTTTGCCTGTGTTGTCAGTAGCATTGAAGATA
GATATCTGAACATTCTTCAAAGCACTACTGTTAGTTGAACACCCACTTTTTTCAATACATCTAATTCATGATATTTTCTCCTTCTGTATGTGCTTGGCTGGCTCTATCAGTCTTGGATGGACATTAGCCA
TAGACTAGGAAAATAAACATCTTATTCAAATGCATTTTAGCTATTAGTAATCAGGAACACCTCTGTAGTCAGGACACCCCATTTTTAAACATCAGGAAGGTGTGAATGACTTGAATTTATAAACTAAA
TAAGCTCGGGAAAGCAGGCATTTCACAAACGGGTGTACAAATAGAGCACACTTGCACATTTTCTACCCAATTAATTTTTGACCAGCGTAGATAAACATGTATTTTGGGACCTTCATGTTGCATGTAGT
CTCCGTTCGCTCATCCGGTGTTCGTCCTCTGTCCACATGTAGGGCTTACTGCTGGGCGAAACCGACCCTTTACCAGGTCGGGGCCAGATGCTCACCCAGACCCCAGGAGTAAGTGAGGCAAATGGC
GTTTATTGCTATAGGCTACGTGTTTAAATACAAGTGTTTCCTCCAATCACGAAGTTACACTTGGCACACACAAGTGGCATAACACACAGGTGGCATAGGAACCTGCACGCGTCACACCTCGTTTCCCT
CGCTACGCCTACAACACACCTCGTTTCCCTCGCTACGCATACAACATCTCCCCCTCCCTATGCCGATAGCTCCCGTCACATCGCGTTCGGAACCTACAGCTGCTCCCTAATTCTATGATATTTTATGCGT
TCATCCATAAACTGATTCATTGTTCTCAATAGACAATCCTGAAAGCACTTGATTATACATGGAATGATAGCTAATAGACAGGCTATGACAAGCAAGGCAAACAGTAGCGTCTTAACACCTTTCGCGA
GCAACCTTCCTAGATCACCAAACGTGCGCGTAAACCAGTCCCCTATGGGATCATCTTCCACCCGTATCTTCTCTGTATGTTCCCTCATGGCTTGGAGCGCCTTGTGAATGGACTCACTGTGATCGCTG
AGATTGAAGCAACACATCCCTTCCACGTCTTGACACCCGTGTCCCTGCGCCAGGAGTAAGAAATCGATGGCTGCTCGATTCTGCAACACTGCGTGCCGGATGCTGCTCGTATCCTCCAGCATCGCAT
TCAATATTAAAGATGTTAAATTTGCTTGCTTAACCGACCAACATGCCAGTCGTTCGATCTCCTTTAAGGCCTGTGCTGCTGCTATACCAGGAGCAAAGAAAGAAGCAAATATCCGGGCTGTCACACTC
CATAGCTGTAGCTCATCACCGCAGTCAGGCGAGAGACGACTCAGCGAGCGCCTGCTACGGCGGTGACCCGTAATGTTCGGCCCATATGTTATCCAGGTGGTAAAGTTAGGAGAGAGCATAGTCAA
TTGTCCTAGATAACAGGGCCCTCCCAAGGCATTACGCGGGATGCCTTGCCAAGCCCTGTCCCCACAAATCAAGAAAATACCTGGTGGGAGTGCCTTAGCAGTCCCATTGTTCCACAACACCTCTGGC
TGTTTCCCGAAGGTAAACCCATATGCATAATAATTCCATTCCGTTGTGCAATTACCACCGCAACCCGTCGTGTTGCTGGAACATTTTGTCCCGTTGCTGTACCCCGCTGACCATTTTCCCCTTTTTGAAC
TACACCAATTAGAAAGGTCCCCTCTATAGTAACGAGTCTCGTTACTGGTGAATCCACAATATCTACGACTTATTGAAAAACTCTCTACATTTATTGGACTTACCATCGTCCATGATTGGTTGACATAGG
GCCTTATAAGAAGGTCATCACTTTTCCATTTTGCTATGAAGTCACGCAATTCTGCTTCCGCCCCACCAGTCCCATTAAAATTCCCATCAAAATTGTGACAGACTCTACTGCGATTGTTCTCTTTATAGCA
CACCGAACCAAAGGTAACACACGTACGTGTTGTTCCGTTCTTGATCATCTGGGACCCTAAAATATCCAATTCTTGGGGGTCCCAAGGGAGGGTTGTATTTAGAGCCTTTATCAAGCATGCTGTTTGG
CTGGCTAAATCGGCATCGTTATCACAAGCAGTAACATTAGTGACGTATCCCTTGAAGGTGTTCAGAGGATACTGTGGAATGCCTATCTGTCGCAAGAAATCCCGCCCCAGAAGAGTTCCGGGGACA
CGCGCCACCAATGGTGTAAGCAACGCCGGTTTCTCTAAGGAGCCATCCCTGTTAATCACAACCACTTGTACCTCATTGGTAGACCGGCGTGAGGGGGTCGCCCCTCCAACCCCCATAATAGACTGCG
AGGCCTCCGCGGGCCACGAGGATGGCCATTCTGTTTCCGTGACCACAGTAACGTCGGCGCCGGAATCTATGAGGGCAGTGACAAAGATAGATAGAGGGCCTTGAAAATCCGGCGGGAGGTTGGT
GCAAGAAATAGTGGCTTTCACCACAGGGCGCTCATGGGTACTTAGAGACAGCGCCGCCATGGGTAAACTTAGGTGAGGGGCCCGCACCGATCGAGAAATGAAGTTGCCGTGCGTGGACGGTCTC
CCGTTGTTATTTCCCTGGCGTGGAGGCCTACGGGCCCTGCATTGTGGTGCTGTATGACCCTTCCGCCCACACTGCTGACATGTTACACCTCCACCCGTGCCCATTCTGCATTGGGCCCTAATATGGCC
CAACTGGCCACATTTAAAGCAAGGTCCTAACTGTCGGTCCGCACTACGATGGTCCGCCTCCCTACAGGCCATCATCACTCCTGCTACCGCCGCAGCTACCTCCCCTGCCACAGACGGACAGGCCTTTT
GCTTATCTAAGACATACTTGATGGCCTCACCAGGGGTTTGGAGCCTCCCCGGCGCCGCTCGCAAAATCCCCTGCACCCCTTCACTGGACTTCTGATACAAGCAATCCAGGATGACGGGACCGTGAAC
CGCTCTGGGCAGATCGGAGCCTTCCACTGCCTTAATTAATCTGTCTGCAAACTCCTGAAACGGCTCTGTGGGGCCTTGCGCGATCTCTGTCCACGGAGTAGTGGGCTCAGCGCTCCTTGCAAGCTTT
CGGAACGCGTCTATTGCAGCGTCCGTAGTTGCTATCAACTCTCCTGGTCTAAGTTGACAAACTTGTGCTTGAGGGGTCAGCAGACCTGGTGCATTGCCCGTTAATCTCTGTATATCGGACCCATATAT
GGGGTTTCTTTGATCACCCTGCGCCGTAATCAGCATTTGTTTTAATTTAGTATGCCACTCCTCTCTCCAGAGCGTATACTGCACTGGTTTCAACACCATGCGCATTAAATTTTCAATATCATAAGGCAG
CATTGGGCCCGATGCTGTGAGGGCCTCAAGGGCATTCATCGTCAATGGCGACTTCAGCCCTTTCTTTTCTACTGCCTCAATCATCCTTGCTACCCCCTTTGGGTCCAACGGGACCCAGATTGGGCCTC
CCTCCTCCGAGACAACAACTGGGAGTGTATAAGTTGCCGCCAGACCTTTCTGTGCCACTTCCTCTTTGATCTGCCCCCAATCAGTAAGCTTAAGTCTCCCCTCCCCCTTTCCTTTTGGGGATGGTCCCG
CCCCCTGCTCCGATGTCGTTGCCAAGGAAGGATATAACGGCTGCTTAGGGTAGGGCGGGGGCGGCGACAGCGCAATAGAATTAGGAGGGGCCGTCGGCGCCGTTGCACCCCCCCCGAGCCTCTG
CGACTCTTCGTTGTCCTTCTCGCAATCTTCCTCTCTCTCCGTCGCCGTTTTATTCGCTGTCGTCTGAGCGCCGGTCTGAGCGCCGGTCTGAGCGCCGGTCTGAGCGCCGGTCTGAGCGCCGGTCCTGC
AAAACTCCAAAGACCCGCTCCCCGGAGCTCCCTCCCCGCTCATGACCGCGCCAAGTTTGTGCTCTGCCCTGGCCGCCTTAAGGGCTCCTAACATCAGTCCCCACGTTTTAAGTTCCGTAGCCTTCTGA
CTGGCCATGGCGCGCTGGCATAGCGCCAAGGTGAGCAAGTCCCACCTACTCGAATCCAAAACGTAGCGGGGAGAGTCCAGCTCCCCCAGCTCATTTAACAGCGACAAAACTGCCGCGATCTCCTTC
CCGGAAGGAGTAGATTTTCCACAGTAGTCCTTACAAAACTGCACAAGTACCTTAATGACTTGGTCCATTGTTACGTATGCGACCTGCCGCCACCTCGCAGGCTCTTAACCGCAATACGTCTTCCGTGC
GCGACCTGCCGCCACCTCACAGGCTCTTCTCCGCGTCACGGGCACGCCGGGCAGCTACCCTCAGCAGACAGATCACGTCGGGGTCACCACTTGTTGCATGTAGTCTCCGTTCGCTCATCCGGTGTTC
GTCCTCTGTCCACATGTAGGGCTTACTGCTGGGCGAAACCGACCCTTTACCAGGTCGGGGCCAGATGCTCACCCAGACCCCAGGAGTAAGTGAGGCAAATGGCGTTTATTGCTATAGGCTACGTGT
TTAAATACAAGTGTTTCCTCCAATCACGAAGTTACACTTGGCACACACAAGTGGCATAACACACAGGTGGCATAGGAACCTGCACGCGTCACACCTCGTTTCCCTCGCTACGCCTACAACACACCTCG
TTTCCCTCGCTACGCATACAACACCTTCAACAGAGGGCAGAACTGGGAGGAGACAAGATTAAAACTGCTTAAACCATAGTTTGTGTTAGAAGTTAATGATTCCTCTACCAAGTACCAACACAGTCTT
GCTTAAGGAGAGAATATTCCAGATTTTGAGTATTCGTGTGGTAGTATAACTTCTGTTGTTGTGGATACAGAACCTAGGTAGGAATCTCTTTAATATTATTTTTGTTTGTCTTAATCTCAAAATGTCCTT
TAGTGTTTGTAGCTAATGAACATTACCTTTGTGGTTTTGTCTCTATTTCCTTTGAACAACTACACTGCTAACATTCATGTTGTAAATATTAATATTTTAGTGCTGATGTTAGAATTTCTATGGGTCTTTCT
GAAAAGTAAACATCATGTATCATGAACAATACAAGTAGCTTTAGGCCTGCATGGCACTATGATGTTCATTTGTCATATTGACTTGACTTGAAAGGTATTACTATGAAACCATAATGTCTTTATGAATC
AACAAGGCGATTGGATTAACACTGCAGTTAATGTAAATCAATACAGTTCCATAAGCTATTAGCTGAAATCTTGATCACATTATTTTTAATATTGTAATCTACTGGATGGTGGTACAGCTGTTGAGAAT
GCAGCTGCAGTGAGAAACAGCCCAAGGGCTTAGTGTCCTTGCTGGACTCTGAAAATTCCTCAGAATCACTTTACTT

Fig. S9 EAV-HP insertion site on chicken chromosome 1 (GenBank accession no: KC632578)
EAV-HP sequence (Araucana); chr.1 chicken genome (Araucana crossbreed); underline= long terminal repeat sequence; underline= target side duplication (TSD)

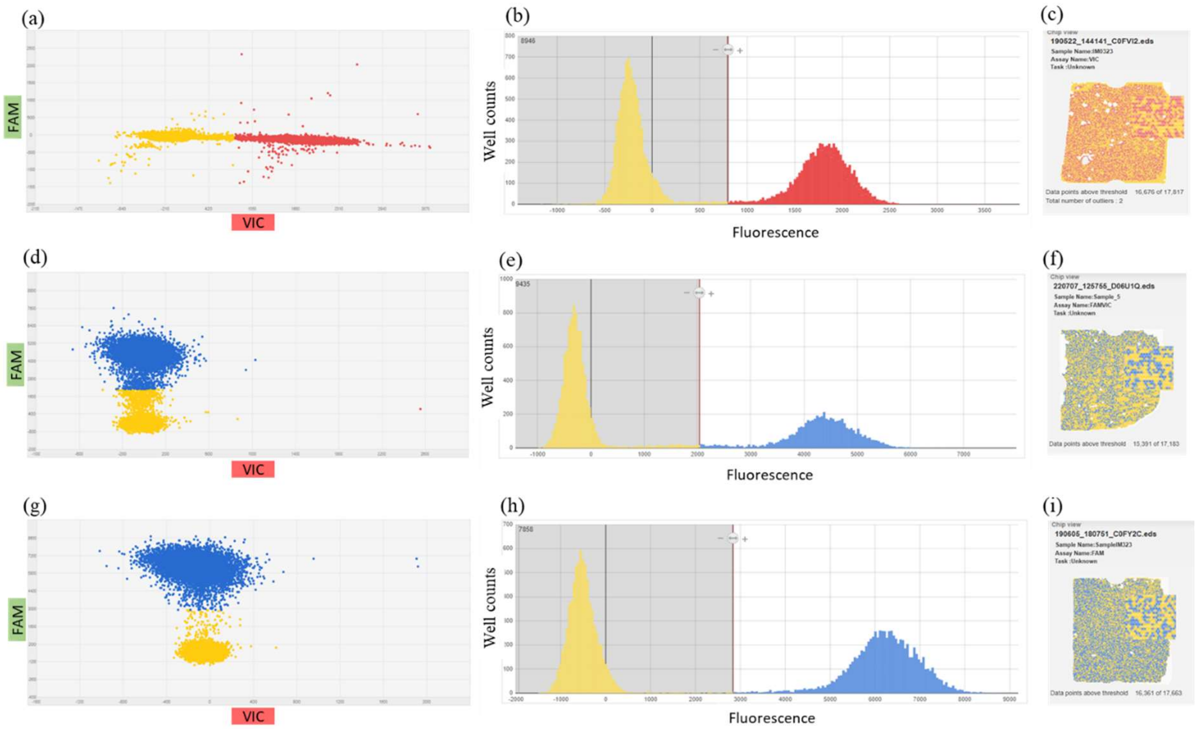

Fig. S10 digital PCR of ß-actin, digital PCR1 target and digital PCR2 target

Scatterplot, histogram and chip view of ß-actin assay (single assay reaction) of homozygous blue-allele bearing PGCs (a, b, c); Scatterplot, histogram and chip view of dPCR1 (single assay reaction) of non-blue allele bearing PGCs (d, e, f); Scatterplot, histogram and chip view of dPCR2 (single assay reaction) of homozygous blue-allele bearing PGCs (g, h, i).

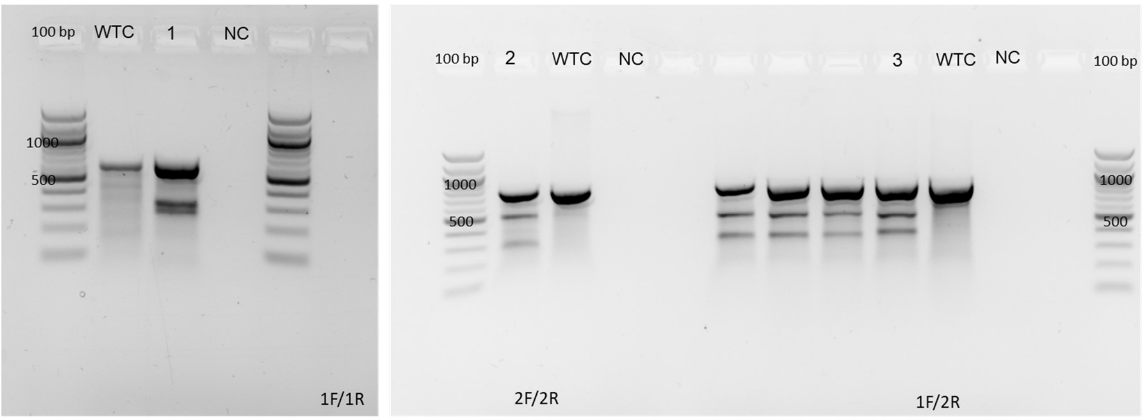

Fig. S11 Uncropped PCR images of Fig.1b

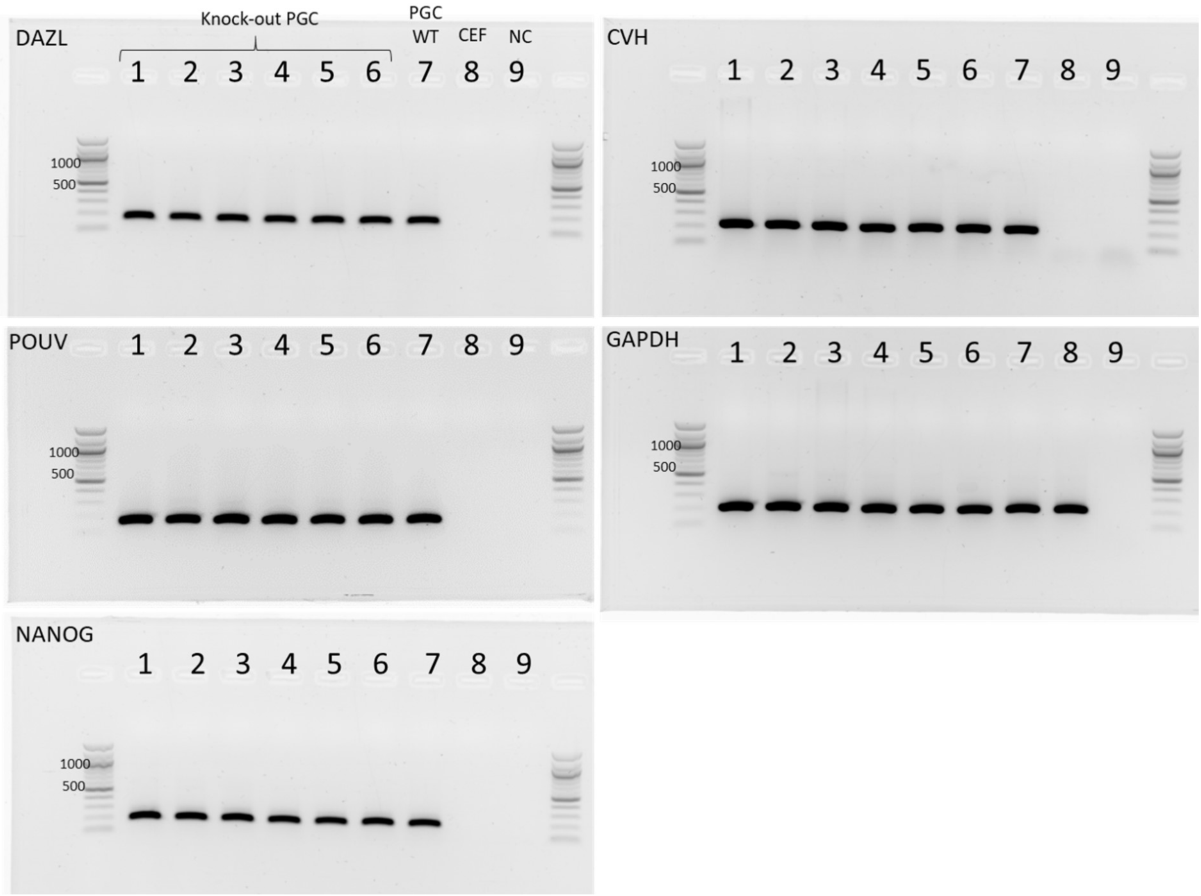

Fig. S12 Uncropped PCR images of Fig. 6a
